# Otic Neurogenesis in *Xenopus laevis*: Proliferation, Differentiation, and the Role of Eya1

**DOI:** 10.3389/fnana.2021.722374

**Published:** 2021-09-20

**Authors:** Suad Hamdan Almasoudi, Gerhard Schlosser

**Affiliations:** School of Natural Sciences, National University of Galway, Galway, Ireland

**Keywords:** Eya1, *Xenopus*, otic vesicle, ear, neurogenesis, delamination, cell polarity, vestibulocochlear ganglion

## Abstract

Using immunostaining and confocal microscopy, we here provide the first detailed description of otic neurogenesis in *Xenopus laevis*. We show that the otic vesicle comprises a pseudostratified epithelium with apicobasal polarity (apical enrichment of Par3, aPKC, phosphorylated Myosin light chain, N-cadherin) and interkinetic nuclear migration (apical localization of mitotic, pH3-positive cells). A Sox3-immunopositive neurosensory area in the ventromedial otic vesicle gives rise to neuroblasts, which delaminate through breaches in the basal lamina between stages 26/27 and 39. Delaminated cells congregate to form the vestibulocochlear ganglion, whose peripheral cells continue to proliferate (as judged by EdU incorporation), while central cells differentiate into Islet1/2-immunopositive neurons from stage 29 on and send out neurites at stage 31. The central part of the neurosensory area retains Sox3 but stops proliferating from stage 33, forming the first sensory areas (utricular/saccular maculae). The phosphatase and transcriptional coactivator Eya1 has previously been shown to play a central role for otic neurogenesis but the underlying mechanism is poorly understood. Using an antibody specifically raised against *Xenopus* Eya1, we characterize the subcellular localization of Eya1 proteins, their levels of expression as well as their distribution in relation to progenitor and neuronal differentiation markers during otic neurogenesis. We show that Eya1 protein localizes to both nuclei and cytoplasm in the otic epithelium, with levels of nuclear Eya1 declining in differentiating (Islet1/2+) vestibulocochlear ganglion neurons and in the developing sensory areas. Morpholino-based knockdown of Eya1 leads to reduction of proliferating, Sox3- and Islet1/2-immunopositive cells, redistribution of cell polarity proteins and loss of N-cadherin suggesting that Eya1 is required for maintenance of epithelial cells with apicobasal polarity, progenitor proliferation and neuronal differentiation during otic neurogenesis.

## Introduction

The otic placode of vertebrates contains a neurosensory domain with neurosensory progenitor pools that give rise to the sensory hair cells of the inner ear responding to vestibular and auditory stimuli as well as to the sensory neurons of the vestibulocochlear ganglion (reviewed in Alsina et al., 2009; Wu and Kelley, 2012; Maier et al., 2014; Alsina, 2020; Elliott et al., 2021; Schlosser, 2021). Because of its central importance for the vertebrate senses of balance and hearing, the generation of sensory hair cells and sensory neurons from the otic vesicle has been described in several vertebrate model organisms, viz. mouse, chick and zebrafish but not in *Xenopus* (Carney and Silver, 1983; Hemond and Morest, 1991; Haddon and Lewis, 1996; Alsina et al., 2004; Raft et al., 2004, 2007; Neves et al., 2007). Moreover, the central roles of the SoxB1 family transcription factors Sox3 and Sox2 for the maintenance of sensorineural progenitors of the inner ear and of the Neurog1 transcription factor for the determination of otic sensory neurons have been elucidated experimentally in these model species (Fode et al., 1998; Ma et al., 1996, 1998, 2000; Andermann et al., 2002; Kiernan et al., 2005; Matei et al., 2005; Neves et al., 2007; Raft et al., 2007; Abelló et al., 2010; Puligilla et al., 2010; Sapede et al., 2012; Evsen et al., 2013; Steevens et al., 2017; Gou et al., 2018).

Whereas Sox2/3 and Neurog1 or the related protein Neurog2 play important roles for neurogenesis not only in the inner ear but also in the central nervous system (Schmidt et al., 2013; Schlosser, 2021), other transcriptional regulators such as the transcription factor Six1 and its coactivator Eya1 are specifically required for the generation of sensory and neuronal cells from the otic vesicle and other placode-derived structures (Xu et al., 1999; Laclef et al., 2003; Zheng et al., 2003; Zou et al., 2004; Bricaud and Collazo, 2006; Schlosser et al., 2008; Ahmed et al., 2012a,b). However, the function of these placode-specific regulators of neurogenesis is much less well understood.

Eya proteins are characterized by a highly conserved C-terminal Eya domain, which serves as a protein-protein binding domain and has tyrosine phosphatase activity, and an N-terminal transactivation domain with threonine phosphatase activity (Ohto et al., 1999; Li et al., 2003; Rayapureddi et al., 2003; Tadjuidje and Hegde, 2013; Rebay, 2016; Roychoudhury and Hegde, 2021). After binding to Six proteins, Eya translocates to the nucleus and acts as a transcriptional coactivator (Ohto et al., 1999; Zhang et al., 2004). Its function as transcriptional coactivator may be modulated by its tyrosine phosphatase activity in some contexts (Li et al., 2003). In addition, Eya protein also binds directly to other proteins and serves as a phosphatase in either the nucleus or the cytoplasm, but these functions are still poorly characterized (Fan et al., 2000; Li et al., 2003; Embry et al., 2004; Cook et al., 2009; Krishnan et al., 2009; Xiong et al., 2009; Ahmed et al., 2012a,b; Merk et al., 2020).

In vertebrates, Eya1 or Eya2 proteins are co-expressed with Six1 during the development of many cranial placodes (precursors of cranial sense organs and ganglia) including the otic placode and the otic vesicle derived from it (Oliver et al., 1995; Abdelhak et al., 1997; Xu et al., 1997; Ohto et al., 1998; Sahly et al., 1999; Pandur and Moody, 2000; David et al., 2001; Laclef et al., 2003; Zheng et al., 2003; Schlosser and Ahrens, 2004; Zou et al., 2004). After Eya1 or Six1 loss of function in mouse and *Xenopus* embryos, cell proliferation in the otic vesicle is reduced (Li et al., 2003; Zheng et al., 2003; Ozaki et al., 2004; Schlosser et al., 2008; Zou et al., 2006, 2008; Chen et al., 2009), while overexpression of high levels of Eya1 and/or Six1 leads to massive proliferation throughout the ectoderm (Kriebel et al., 2007; Schlosser et al., 2008).

Furthermore, loss of function of Eya1 or Six1 in mice, zebrafish and *Xenopus* inhibits the expression of transcription factors promoting neuronal and sensory (hair cell) determination and differentiation (e.g., Atoh1, Neurog1, Neurog2, NeuroD1) in the otic vesicle, thereby blocking neuronal and sensory differentiation (Xu et al., 1999; Laclef et al., 2003; Zheng et al., 2003; Zou et al., 2004; Bricaud and Collazo, 2006; Schlosser et al., 2008; Ahmed et al., 2012a,b), while overexpression of low levels of Six1 and Eya1 instead leads to ectopic differentiation of sensory neurons and hair cells (Schlosser et al., 2008; Ahmed et al., 2012a,b).

Taken together, this indicates that Eya1 and Six1 play central roles for both progenitor proliferation and neuronal/sensory differentiation in the developing otic vesicle. This dual role of Eya1 and Six1 appears to be mediated at least in part by the cooperative action of Eya1 and Six1 in a protein complex, which directly transcriptional activates genes involved in progenitor maintenance and proliferation (e.g., Sox2/3, Hes5) as well as genes promoting neuronal or sensory differentiation (e.g., Neurog1, Atoh1, POU4f1, Islet2) (Ahmed et al., 2012a,b; Riddiford and Schlosser, 2016; Li et al., 2020). Previous studies have suggested that the level of Eya1 and Six1 may be important in determining whether it promotes one or the other function, with high levels promoting progenitors and low levels promoting differentiation (Schlosser et al., 2008; Zou et al., 2008; Riddiford and Schlosser, 2017). However, the underlying mechanism is currently unknown.

Furthermore, recent evidence suggests that Eya1 may also affect the balance between proliferating progenitors and differentiating neurons in Six1-independent ways and possibly by acting in the cytoplasm. In the cerebellum, Eya1 has been shown to directly dephosphorylate cell polarity proteins such as atypical protein kinase C (aPKC), thereby affecting apicobasal cell polarity and changing the balance between proliferating and differentiating cells by controlling the proportion between symmetric and asymmetric cell divisions (Merk et al., 2020). Another study (El Hashash et al., 2011) reported a similar role of Eya1 in the lung epithelium but was subsequently retracted (El Hashash et al., 2017).

To begin to unravel the mechanism by which Eya1 affects progenitor development and neuronal differentiation in the developing inner ear, the existing data on *Eya1* mRNA expression are not sufficient. In addition, we need to gather detailed information on the subcellular localization of Eya1 proteins, their levels of expression as well as their distribution in relation to progenitor and neuronal differentiation markers.

The aim of the present study is to provide the first detailed description of otic neurogenesis in *Xenopus* with a special emphasis on the distribution and function of Eya1, thereby paving the way for further functional studies in the frog. We have characterized the spatiotemporal pattern of otic neurogenesis using immunostaining for markers of basal lamina (laminin), apically localized cell polarity proteins (aPKC, Par3, phosphorylated Myosin light chain and N-cadherin), cell proliferation (pH3 and EdU incorporation), progenitor cells (Sox3) and differentiating neurons (Islet1/2, acetylated tubulin). This allowed us to elucidate (1) the spatiotemporal pattern of otic neurogenesis as judged by the delamination of neuroblasts through gaps in the basal lamina; (2) the organization of the neurogenic otic epithelium including the subcellular distribution of cell polarity proteins; and (3) the changing distribution of cell proliferation, neurosensory progenitors, and differentiating neurons during development of the otic vesicle.

To characterize the subcellular localization of Eya1, as well as its levels and distribution during the period of neurogenesis in the otic vesicle, we have used a specific antibody raised against *Xenopus* Eya1 and confocal microscopy. Finally, we demonstrate in loss and gain of function experiments, that Eya1 is required for proliferation, progenitor maintenance and neuronal differentiation in the otic vesicle and vestibulocochlear ganglion and for the proper distribution of cell polarity proteins and N-cadherin. Additional functional studies will be needed to clarify, which of these functions is mediated by transcriptional regulation in the nucleus (probably in cooperation with Six1) or by the phosphatase activity of Eya1 acting in the cytoplasm or nucleus.

## Materials and Methods

### Expression Constructs

*GR-Eya1* and *GR-Six1* mRNAs were made from pCS2+-GR-myc-Eya1α and pCS2+-GR-myc-Six1 plasmids (Schlosser et al., 2008), respectively. Myc-tagged Eya1 mRNA (myc-Eya) was made from pCS2+-myc-Eya1α (Schlosser et al., 2008). pCS2+-Eya1α (Ahrens and Schlosser, 2005) and pDH105-Six1 (Pandur and Moody, 2000) were used for *in vitro* transcription and translation reactions and subsequent detection in western blots. The *mGFP* mRNA encoding a membrane-tethered from of GFP was made from MemGFP-pCS2+ plasmid (containing the ras membrane-localization (CAAX) sequence fused to the carboxy terminus of GFP; kindly provided by J. Wallingford) (Moriyoshi et al., 1996; Wallingford et al., 2000).

### Morpholinos

Two different Morpholino antisense oligonucleotides (MO) blocking translation of Eya1 were coinjected for Eya1 knockdown: Eya1MO1 (5′-TACTATGTGGACTGG TTAGATCCTG-3′) targets base pairs 10 to 34 of the Eya1.L coding region and also is complementary to base pairs 10 to 34 of Eya1.S with two mismatches, whereas Eya1MO2 (5′-ATATTTGTTCTGTCAGTGGCAAGTC-3′) is specifically directed against base pairs −7 to −31 in the 5′ UTR of Eya1.L. The efficacy and specificity of these MOs was previously verified (Schlosser et al., 2008) and confirmed here in western blots following *in vitro* transcription and translation (TNT-coupled reticulocyte lysate kit, Promega) of pCS2+-Eya1α (1 μg/25 μl reaction) with and without MO (25 μM) using an guinea pig anti *Xenopus*-Eya1 antibody (anti-Eya1 GP1) as previously described (Ahrens and Schlosser, 2005). For control experiments, a standard control MO (5′CCTCTTACCTCAGTTACAATTTATA-3′, Schlosser et al., 2008) was used.

### Microinjections

Embryos of *Xenopus laevis* were staged according to Nieuwkoop and Faber (1967) and injected according to standard procedures (Sive et al., 2000). Capped mRNAs were synthesized with Message Machine Kit (Ambion) and injected into single blastomeres at the 2- to 4-cell stage that give rise to the dorsal ectoderm. Unless otherwise noted, the following amounts of mRNAs were injected: *mGFP*: 250 pg; *GR–Eya1*: 500 pg; *GR–Six1*: 500 pg. Morpholinos (see above) were injected singly or as a cocktail (1–10 ng each) into single blastomeres at the 2–4 cell stage. *mGFP* was coinjected as lineage tracer to identify the injected side. For activation of hormone-inducible constructs, embryos were incubated in dexamethasone (DEX; 10 μM; Sigma) from stages 16–18 onward until they reached the stage for fixation. In a previous study, we confirmed that these GR-fusion constructs function comparable to wild-type protein, that embryos injected with GR-constructs and raised in the absence of DEX showed minimal effects and that DEX treatment alone does not significantly alter gene expression (Schlosser et al., 2008) in accord with published accounts of this method (Hollenberg et al., 1993; Mattioni et al., 1994; Kolm and Sive, 1995; de Graaf et al., 1998).

### Immunohistochemistry and *in situ* Hybridization

All immunohistochemical experiments were done in at least five embryos per marker analyzed; functional studies followed by immunostaining were done in at least three embryos per marker analyzed. Embryos were fixed with 4% paraformaldehyde (PFA) in phosphate buffer (PB) overnight at 4°C. Following two washes in PB (10′ each), embryos were rinsed in 50% EtOH (5′) and stored in 70% EtOH at 4°C. Embryos destined for N-cadherin immunostaining were instead fixed and stored in Dent’s fixative (80% methanol, 20% dimethyl sulfoxide). Embryos were then rehydrated and cryosectioned (10 μm) followed by immunohistochemistry on sections as previously described (Schlosser and Ahrens, 2004). The primary antibodies used and their dilutions are summarized in Table 1. Sox3 was revealed using a polyclonal rabbit anti-Sox3 antibody recognizing *Xenopus* Sox3 (Zhang et al., 2003). Eya1 was revealed using the guinea pig anti-*Xenopus*-Eya1 antibody (anti Eya1 GP1) raised against the peptide RLSGSGDSPSGTGLDNSHINS corresponding to amino acids 12–32 of *Xenopus* Eya1 (Ahrens and Schlosser, 2005).

**TABLE 1:** Primary antibodies used in this study.

Antigen	Supplier, Product number	Species	Dilution
Laminin	Sigma, L9393	Rabbit-Polyclonal	1:25
Proliferating cell nuclear antigen (PCNA)	Dako, Carpinteri (CA), M 879	Mouse-Monoclonal (clone PC10)	1:500
Phospho-Histone 3 (pH3)	Merck, 06-570	Rabbit-Polyclonal	1:100
GFP	Santa Cruz Biotechnology, sc-8334	Rabbit-Polyclonal	1:1000
GFP	Abcam, 9F9.F9	Mouse-Monoclonal	1:1000
Tubulin, Acetylated	Sigma, T6793	Mouse-Monoclonal	1:1000
N-cadherin (CDH2)	Abnova, PAB7876	Mouse-Monoclonal	1:200
Myosin light chain (phosph S20)	Abcam, ab2480	Rabbit-Polyclonal	1:200
Atypical protein kinase (aPKC)	Santa Cruz Biotechnology, sc-216	Rabbit-Polyclonal	1:200
Partitioning-defective 3 (PAR3)	Millipore, o7-330	Rabbit-Polyclonal	1:200
Eya1	(Ahrens and Schlosser, 2005)	Guinea pig-Polyclonal	1:50
Sox3	(Zhang et al., 2004)	Rabbit-Polyclonal	1:250
Islet-1/2	Developmental Studies Hybridoma Bank (DSHB), 39.4D5	Mouse-Monoclonal (clone 39.4D5)	1:200

After overnight incubation in primary antibodies, PCNA immunohistochemistry followed published protocols (Wullimann et al., 2005). For all other primary antibodies, except for those revealed by tyramid signal amplification (see below), overnight incubation in primary antibodies (up to 2 days for anti Sox3) was followed by incubation for at least 2 h in Alexa488- or Alexa594-conjugated anti-mouse (Molecular Probes A11001 or A11005, respectively) or in Alexa488- or Alexa594-conjugated anti-rabbit secondary antibodies (Molecular Probes A11008 or A11012, respectively) diluted 1:500 and supplemented with DAPI (100 ng/μl) followed by final washes and coverslipping with Fluoroshield (Sigma: F6182). For double-immunostaining, both primary antibodies raised in different species were applied simultaneously, as were the two secondary antibodies.

To reveal Par3, phosphorylated myosin light chain (MLC), aPKC, Eya1 and Islet1/2, we used a tyramide signal amplification (TSA) kit (TSA plus Cyanine 3/Fluorescein System, Perkin Elmer: NEl753001kT) according to the manufacturers instruction using HRP-coupled goat anti-rabbit antibody (Invitrogen G56120, 1:500) to reveal anti-MLC, anti-aPKC and anti-Islet1/2 and HRP-coupled goat anti-guinea pig (Abcam 6771,1:500) to reveal Eya1. For double-immunostaining of Eya1 or Islet1/2 with Sox3, Eya1 or Islet1/2 were revealed first using the TSA kit. Then, slides were immersed in boiling sodium citrate solution (10 mM, pH = 6) for 8 min to strip all antibodies used to detect the first antigen. During this procedure, the insoluble fluorophore-tyramide deposition is retained on the tissue (Toth and Mezey, 2007; Francisco-Cruz et al., 2020; Lyu et al., 2020). Slides were then stained with anti-Sox3 antibodies in a second step, following the regular protocol. Non-specific binding of secondary antibodies was not observed when the primary antibody was omitted in control reactions. For peptide competition assays, the peptide against which the Eya1 antibody was raised was applied together with the Eya1 antibody (5 μg peptide/1 μg Eya1 antibody). Sections were analyzed with a regular compound microscope and by confocal microscopy (Olympus Fluoview 1000 Confocal Microscope).

Wholemount *in situ* hybridization was carried out as previously described (Schlosser and Ahrens, 2004) using digoxigenin-labeled antisense probes for *Neurog1* (Nieber et al., 2009), *Sox3* (Penzel et al., 1997), *Sox2* (De Robertis et al., 1997), and *Atoh1* (Riddiford and Schlosser, 2016). After *in situ* hybridization, vibratome sections (40 μm) were cut and Sox3 was revealed immunohistochemically as described above.

### EdU Incorporation and Staining

To label the entire population of proliferating cells, embryos were incubated in a 4 mM solution of 5-ethynyl-20-deoxyuridine (EdU) for 16 h at 15°C until they reached stages 26, 28, or 35 when they were fixed in 4% PFA. EdU was then revealed on cryosections with Click-iT Plus EdU Alexa Fluor 594 and 488 Imaging Kit (Thermo Fisher Scientific) following the manufacturer’s instructions. If combined with immunostaining, the immunostaining protocol was completed first. After incubation with the secondary antibody, each slide was washed twice with 3% BSA in PBS for 30′ at room temperature, followed by the EdU reaction.

## Results

### Time Course of Neuronal Delamination and Differentiation

In *Xenopus*, the otic vesicle begins to invaginate from the posterior placodal area at stage 22/23 and has completely separated from the surface ectoderm by stage 28 (Schlosser and Northcutt, 2000; Schlosser and Ahrens, 2004). Whereas a previous study has reported that neurons of the vestibulocochlear ganglion first differentiate at stage 31 (Quick and Serrano, 2005), a detailed schedule of neuronal delamination and differentiation has not yet been described.

We, therefore, used immunostaining for laminin to determine, when neurons start to delaminate from the otic vesicle through breaches in the basal lamina. Immunostaining for acetylated tubulin was used to determine, when neurons are differentiated and begin to send out neurites. To visualize the cells in the otic epithelium, we acquired z-stacks of confocal images after injection of *mGFP* into early *Xenopus* embryos and labeling of nuclei by DAPI staining. For some embryos, we reconstructed cell shapes manually by tracing and superimposing the mGFP stained cell membranes from multiple confocal planes.

**FIGURE 1:**
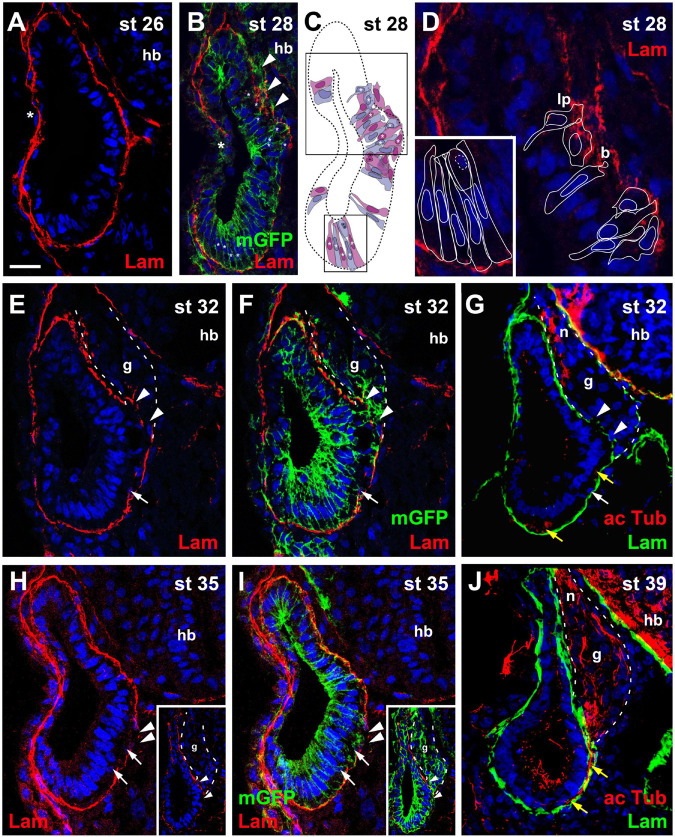
Time course of neurogenesis and neuronal migration in the otic vesicle. Immunostaining for laminin (Lam) in transverse sections through the center of the left otic vesicle of *Xenopus* embryos from stage 26 to 39 analyzed in single confocal planes **(A–G)** or maximum intensity projections of z-stacks **(H–J)** (dorsal to the top, medial to the right). Some sections have also been immunostained for acetylated tubulin **(G,J)** or a membrane bound form of GFP (mGFP) following *mGFP* mRNA injection **(B,F,I)**. DAPI was used to label nuclei. Different channels of same section shown in **(E,F)** and in **(H,I)**. Arrowheads indicate breaches in the basal lamina. b, blob; g, vestibulocochlear ganglion (outlined with hatched lines); hb, hindbrain; lp, lamellipodium; n, vestibulocochlear nerve. **(A)** At stage 26 the otic vesicle has largely invaginated and is surrounded by a basal lamina. Reorganization of the basal lamina takes place where otic epithelia are in the process of fusion laterally (asterisk). **(B–D)** At stage 28 the first breaches appear in the basal lamina on the medial side of the otic vesicle (arrowheads), whereas reorganization of the basal lamina continues laterally (asterisk). **(C)** Shows cell shapes reconstructed from mGFP staining of a z-stack, from which **(B)** was taken. Cells are shown in alternating blue and purple colors for clarity. They form a single-layered, pseudostratified epithelium. Outlines of cells marked with asterisks in the black boxes in **(C)** are shown at higher magnification in **(D)** superimposed on laminin staining. Laminin is displaced where lamellipodia protrude from cells which probably migrate out of the otic vesicle. Some cells form blob-like protrusions through gaps in the basal lamina. **(E–J)** At later stages (stages 32–39) the ganglion (g; outlined with white hatched line in **(E–G)** and axons of the vestibulocochlear nerve (n) can be recognized between the otic vesicle and the hindbrain. **(H,I)** Show a section through the stage 35 otic vesicle immediately posterior to the main body of the ganglion, while a section through another otic vesicle at the center of the ganglion is shown in insets. At stages 32 **(E–G)** and 35 **(H,I)** there are still medial gaps in the basal lamina next to the ganglion, but these have closed by stage 39 **(J)**. Cells on the ventromedial side of the otic vesicle (white arrows) and acetylated tubulin stained axons (yellow arrows) located between the otic epithelium and the basal lamina are indicated. Scale bar in **(A)**: 25 μm (for all panels).

At stage 26, in most embryos the otic vesicle is completely surrounded by a basal lamina except for the central part of its lateral region, where the dorsal and ventral rim of the invaginating vesicle are still in the process of fusion (Figure 1A). At stage 28, while the process of fusion and reorganization of the basal lamina is still ongoing laterally, new gaps appear in the basal lamina on the medial side of the otic vesicle approximately in the middle of its dorso-ventral extension. Laminin distribution in this area appears fragmented and disorganized. Labeling of cell membranes of otic epithelial cells by injection of *mGFP* and reconstruction of cell shapes from z-stacks of confocal images revealed that the irregularities of laminin distribution in this area correspond to protrusions of otic epithelial cells (Figures 1B–D). Some cells, which still form part of the otic epithelium extend small blob-like basal protrusions through small perforations in the basal lamina, suggesting that the perforations may have been generated by mechanical or chemical action of the protrusions as previously suggested in mouse and chick embryos (Meier, 1978; Carney and Silver, 1983; Hemond and Morest, 1991). Other cells with broad and lamellipodia-like basal protrusions typically have only small and slender extensions on their apical sides, which reach into the otic epithelium without extending to the lumen of the otic vesicle. In addition, their nuclei tend to be located on the basal side of the otic epithelium. Taken together, this indicates that these cells are in the process of delamination from the otic vesicle.

At stages 32, 33/34, and 35, the basal lamina continues to be disrupted and cells continue to delaminate in this central portion of the medial otic epithelium. Delaminated cells congregate to form the vestibulocochlear ganglion in the region between the dorsal half of the otic vesicle and the hindbrain (Figures 1E–I). Immunostaining with acetylated tubulin reveals that the first neurites extend from the ganglion at stage 32 (Figure 1G). At stage 39, the basal lamina has reformed on the medial side of the otic epithelium, suggesting that delamination of cells has largely stopped (Figure 1J).

At stages 32–39, cells and neurites immunopositive for acetylated tubulin, are also observed on the ventral and ventromedial side of the otic vesicle sandwiched between the otic epithelium and the basal lamina (Figures 1E–J). There are no breaches of the basal lamina in this area suggesting that cells delaminating on the ventral side of the otic vesicle migrate dorsally on the inside of the basal lamina before joining the ganglion and that the same course is subsequently taken by their dendrites.

Taken together, our observations suggest that neuronal delamination from the otic vesicle mainly occurs from stage 27 (possibly starting at late stage 26 in some embryos) to stage 39 although minor contributions at later time points cannot be ruled out. The first neurites grow out at stage 32 indicating that the first neurons have differentiated at that time. After identifying this time window, we next sought to further characterize the phenotype and distribution of the neuronal progenitor cells in the otic epithelium.

### The Otic Epithelium Is a Pseudostratified Epithelium With Apicobasal Polarity

**FIGURE 2:**
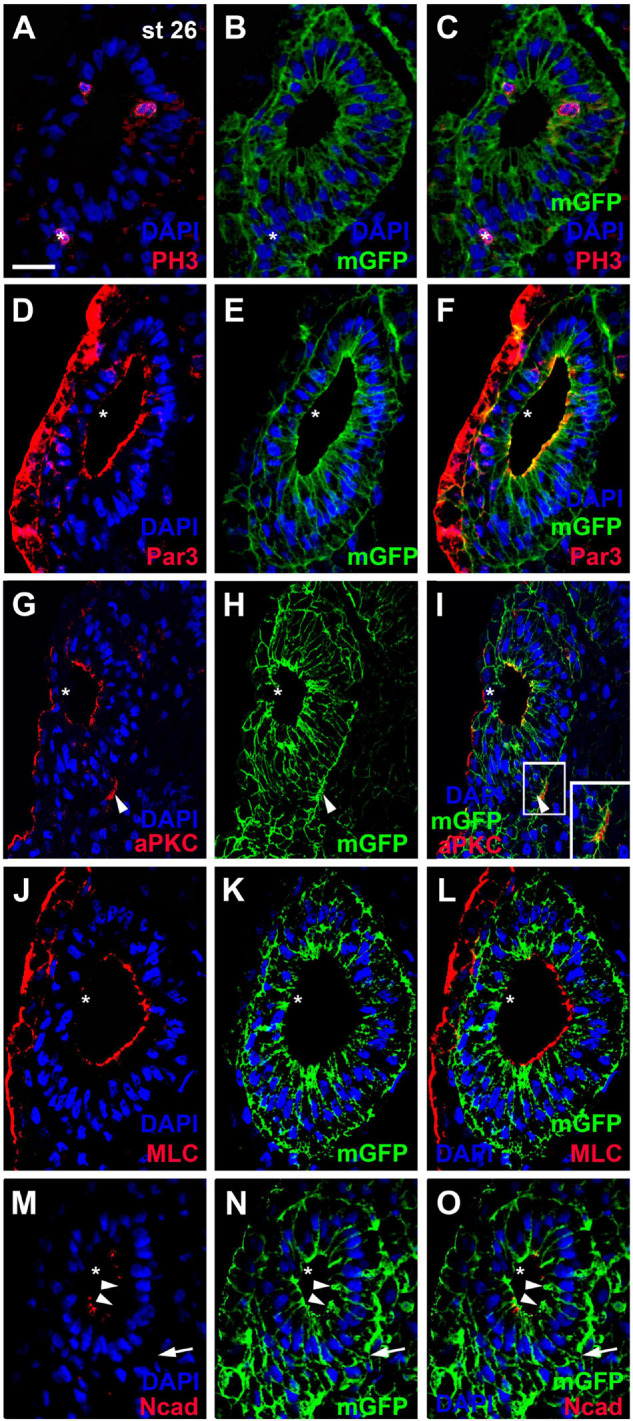
Distribution of mitoses and apico-basal markers in the otic epithelium at stage 26. Transverse sections through the center of the left otic vesicle of *Xenopus* embryos at stage 26 analyzed in single confocal planes (dorsal to the top, medial to the right). Sections have also been immunostained for membrane GFP (mGFP) following *mGFP* mRNA injection. DAPI was used to label nuclei. Different channels of same section shown in **(A–C)**, **(D–F)**, **(G–I)**, **(J–L)**, and **(M–O)**. **(A–C)** Mitotic, pH3 positive cells in the otic epithelium (asterisks) are located near the apical (luminal) surface. One pH3 positive nucleus (asterisk) is located outside the invaginated otic vesicle in the adjacent posterior placodal area, which is in a process of dynamic reorganization where apical and basal surfaces cannot be ascertained. **(D–O)** Immunostaining for cell polarity proteins Par3 **(D–F)**, aPKC **(G–I)**, MLC **(J–L)**, and N-cadherin (Ncad; **(M–O)**). Note the prevalence of apical and/or apicolateral staining. Apical/apicolateral staining is notably absent from the lateral domain of the otic epithelium, where invaginating epithelia fuse (asterisks in **(D–O)**). In addition to its distribution on the apical side of the otic vesicle, aPKC is localized to basal protrusions on the ventromedial side of the otic epithelium (white arrowheads in **(G–I)**). White boxed area in **(I)** is shown at higher magnification in insets. Apicolateral Ncad-staining is markedly reduced in the medial and ventromedial otic epithelium (arrowheads), where cells begin to form basal protrusions (arrow). Scale bar in **(A)**: 25 μm (for all panels).

**FIGURE 3:**
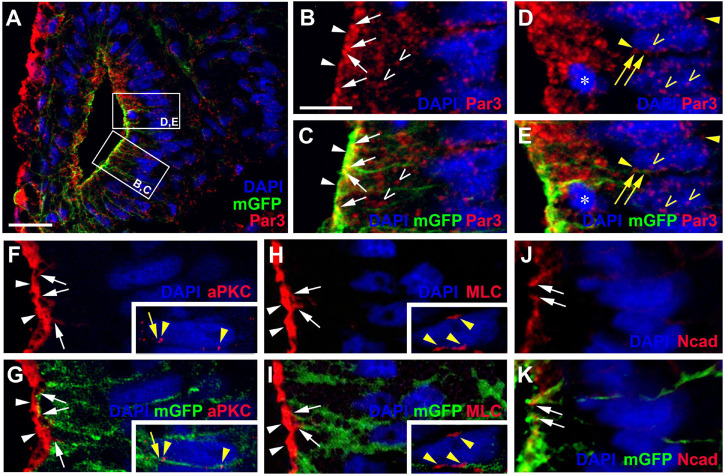
Apico-basal polarity in the pseudostratified otic epithelium at stage 26. Immunostaining for the cell polarity proteins Par3, aPKC, MLC, and N-cadherin (Ncad) in transverse sections through the center of the left otic vesicle of *Xenopus* embryos at stage 26 analyzed in single confocal planes (dorsal to the top, medial to the right). Sections have also been immunostained for membrane GFP (mGFP) following *mGFP* mRNA injection. Distribution of cell polarity proteins in medial otic epithelium as indicated in overview (**(A)**; Par3) and higher magnified views of Par3 (lower and upper box in **(A)** shown in **(B–E)**), aPKC **(F,G)**, MLC **(H,I)**, and N-cadherin **(J,K)**. DAPI was used to label nuclei. Different channels of same region shown in **(B,C)**, **(D,E)**, **(F,G)**, **(H,I)** and **(J,K)**. Inserts in **(F–I)** show nuclei from adjacent cells in medial otic epithelium, which show clear perinuclear and membrane staining. All proteins are localized to the apical (Par3, aPKC, MLC) and/or apicolateral (Par3, aPKC, MLC, Ncad) surface of cells. In addition, Par3 is localized to some cytoplasmic regions and nuclei and Par3, aPKC, and MLC show staining of membranes and perinuclear staining associated with some nuclei. White arrowheads indicate apical staining; white arrows: apicolateral (junctional) staining; white open arrowheads: cytoplasmic staining; yellow arrows: membrane staining next to nuclei; yellow arrowheads: perinuclear staining; yellow open arrowheads: nuclear staining; asterisk in **(D,E)**: dividing nuclei. Scale bars **(A)** 25 μm; **(B)** 10 μm (for **(B–K)**).

Our reconstructions of cell shapes from z-stacks revealed that the otic epithelium at stages 26 and 28 is a monolayered epithelium of columnar cells, which are higher on the medial than on the lateral side (Figures 1A–D, 2A–F). Nuclei are, however positioned at different levels along the apicobasal axis of cells, resulting in a pseudostratified appearance of the epithelium. Mitotic nuclei as revealed by pH3 immunostaining or DAPI-labeling of condensed chromosomes in dividing cells, were always localized apically, i.e., near the lumen of the otic vesicle (Figures 2A–C, 3A,D,E). This suggests that nuclei in the *Xenopus* otic epithelium probably undergo interkinetic nuclear migration from an apical position at mitosis to a more basal position during the S-phase of the cell cycle and back as described for the neuroepithelium and some placodes in other vertebrates (Sauer, 1936; Spear and Erickson, 2012; Alsina and Whitfield, 2017).

To assess whether cells in the otic epithelium are polarized along their apicobasal axis, we analyzed the distribution of the apical polarity proteins Partition defective 3 (Par3), atypical protein kinase C (aPKC) and phosphorylated myosin light chain (MLC) using immunostaining and confocal microscopy. Par3 and aPKC, together with another Par family member, Par6, are known to localize to the apical membrane, where they form the Par complex with a central role in the establishment and maintenance of apicobasal epithelial cell polarity (reviewed in Knoblich, 2010; Chen and Zhang, 2013; Vorhagen and Niessen, 2014; Hapak et al., 2018). MLC also localizes apically where it is involved in the regulation of tight junctions and interacts with the Par complex in some epithelia (Turner et al., 1997; Yu et al., 2015). At stage 26, all three proteins were found associated with the apical and apicolateral cell membranes throughout the otic epithelium except for the lateral side, probably reflecting the ongoing invagination of the otic vesicle and reorganization/fusion of the otic epithelium on the lateral side (Figures 2D–L, 3A–I). Furthermore, the cell adhesion molecule N-cadherin was found localized to the apicolateral cell membranes (Figures 2M–O, 3J,K), where it is probably associated with adherens junctions as in other epithelia (Miyamoto et al., 2015). However, apicolateral N-cadherin is not observed in the lateral part of the otic vesicle, where the epithelia of the invaginating otic vesicle fuse. It is also reduced in a medial and ventromedial domain, where otic epithelial cells have formed basal protrusions suggesting that downregulation of N-cadherin in adherens junctions precedes cell delamination from the otic vesicle (Figures 2M–O).

In addition to its localization to the apical/apicolateral cell membrane, at stage 26 Par3 immunostaining was also observed in a granular cytoplasmic pattern, in particular near the apical side, and in multiple small spots in the nuclei of otic epithelial cells (Figures 3A–E). Strikingly, multiple Par3-immunopositive spots were also found attached to the outside of many nuclei and these were often closely associated with separate spots of Par3 in the adjacent cell membrane (Figures 3D,E). Similar patterns of perinuclear staining were observed for aPKC and MLC (Figures 3F–I).

**FIGURE 4:**
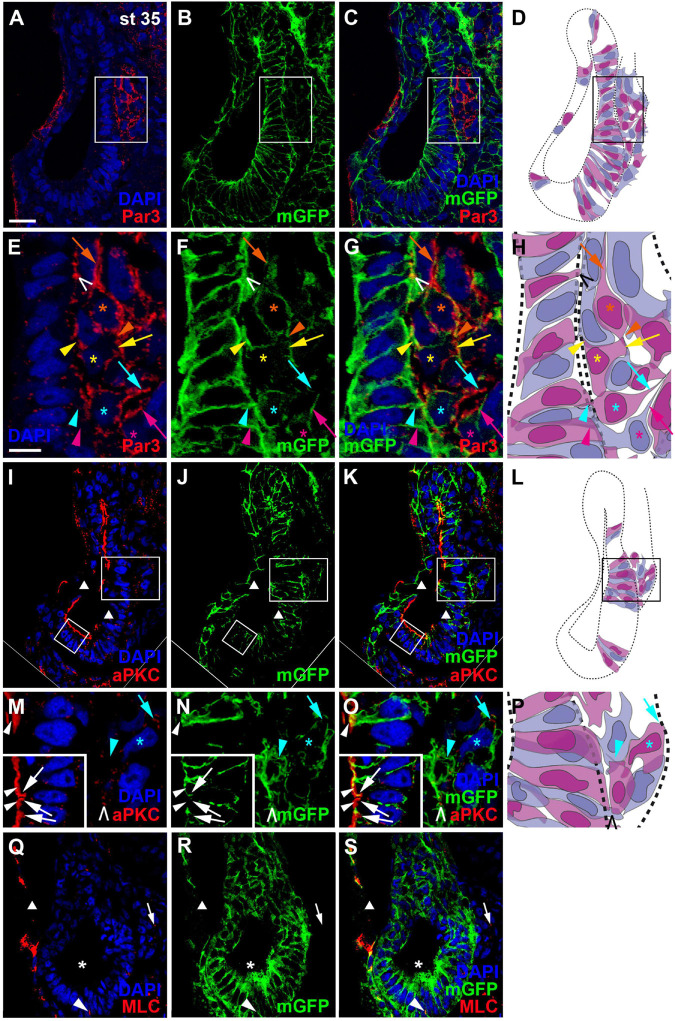
Distribution of apico-basal polarity markers in otic vesicle and vestibulocochlear ganglion at stage 35. Immunostaining for the cell polarity proteins Par3 (**(A–C)**, **(E–G)**), aPKC (**(I–K)**, **(M–O)**) and MLC **(Q–S)** in transverse sections through the center of the left otic vesicle of *Xenopus* embryos at stage 35 analyzed in single confocal planes (dorsal to the top, medial to the right). Sections have also been immunostained for membrane GFP (mGFP) following *mGFP* mRNA injection. DAPI was used to label nuclei. Different channels of same section and cell shapes (shown in alternating blue and purple colors for clarity) reconstructed from mGFP staining of a z-stack are shown in **(A–D)**, **(I–L)**, and **(Q–S)** with boxed regions shown magnified in **(E–H)** and **(M–P)**. **(A–H)** Par3 staining at stage 35 is strongly reduced in the otic epithelium and is mostly found localized to basal protrusions and to the membranes of vestibulocochlear ganglion cells. **(I–O)** aPKC is still localized to the apical side of the otic epithelium at stage 35, but is also enriched in membranes of basal protrusions and the leading edge of delaminating ganglion cells. **(Q–S)** MLC immunostaining at stage 35 is strongly reduced in the apical and apicolateral part of otic epithelial cells (single asterisks), but some staining is found in lateral cell membranes (arrowhead) and in the cytoplasm of vestibulocochlear ganglion cells (arrow). White arrowheads indicate apical staining; white arrows: apicolateral (junctional) staining; white or black open arrowheads: basal protrusions; colored arrows: leading edge (axon forming) of vestibulocochlear ganglion cells; colored arrowheads: trailing edge (dendrite forming) of vestibulocochlear ganglion cells; colored asterisks: nuclei. Individual cells are indicated by different colors. Triangles indicate imaging artifacts (absence of signal in single confocal plane due to bends in section). Scale bars **(A)** 25 μm (for **(A–D)**, **(I–L)**, **(Q–S)**); **(E)** 10 μm (for **(E–G)**, **(M–O)**).

Once cells have started to delaminate from the otic epithelium, there are some notable changes in the distribution of cell polarity proteins. We started to observe this for aPKC already in a few stage 26 embryos, in which aPKC was localized to basal protrusions from epithelial cells in the ventromedial otic epithelium in addition to its apical distribution (Figures 2G–I). This suggests that delamination from the otic vesicle starts at late stage 26 or stage 27. At stage 35, the distribution of all cell polarity proteins in the otic vesicle has changed. Both Par3 and aPKC proteins now are also found localized to the membrane in basal protrusions of delaminating cells (Figure 4). Moreover, Par3 immunostaining in otic epithelial cells (including their apical and apicolateral membranes) is strongly reduced, whereas strong Par3-immunoreactivity is found throughout the membranes of vestibulocochlear ganglion cells (Figures 4A–H) including their leading edge, which is directed toward the hindbrain (where the axon will form), and their trailing edge, which is directed toward their region of origin from the otic vesicle (where the dendrite will form). In contrast, aPKC maintains its localization to apical and apicolateral membranes of otic epithelial cells at stage 35, with additional localization to the leading edge of ganglion cells (Figures 4I–P). MLC also has become strongly reduced on the apical and apicolateral side of otic epithelial cells at stage 35 similar to Par3, while weak MLC staining remained in some lateral membranes of otic epithelial cells (Figures 4Q–S). There was also weak MLC staining in the cytoplasm of vestibulocochlear ganglion cells (Figures 4Q–S). Taken together this suggests that apart from their early role in maintaining apicobasal polarity in the otic epithelium, cell polarity proteins, in particular Par3 and aPKC, may play additional roles for the delamination, migration and possibly neurite extension in the otic neuroblasts that will form the vestibulocochlear ganglion cells as discussed in more detail below.

### Proliferation and Distribution of Progenitor Cells

To gain insights into the early stages of otic neurogenesis, we next studied the distribution of cell proliferation and of sensorineural progenitors in the developing otic vesicle. To quantify the proportion of mitotic cells, we analyzed the proportion of DAPI-positive nuclei in the otic vesicle, which are immunostained for the mitotic marker pH3. At stage 26, mitotic cells comprised 1.63 ± 0.51% of otic epithelial cells with a slight but not quite significant increase to 2.6 ± 0.39% at stage 35 (*p* = 0.057, *t*-test, Supplementary Table 1). To label the entire population of proliferating cells, we sacrificed embryos immediately after a 16 h incubation in EdU. We also used PCNA-immunostaining to label proliferating cells in the S-phase of the cell cycle, thereby marking the majority of proliferative cells. Sensorineural progenitors were visualized by immunostaining for Sox3 (Neves et al., 2007; Schlosser et al., 2008; reviewed in Pevny and Placzek, 2005; Sarkar and Hochedlinger, 2013).

**FIGURE 5:**
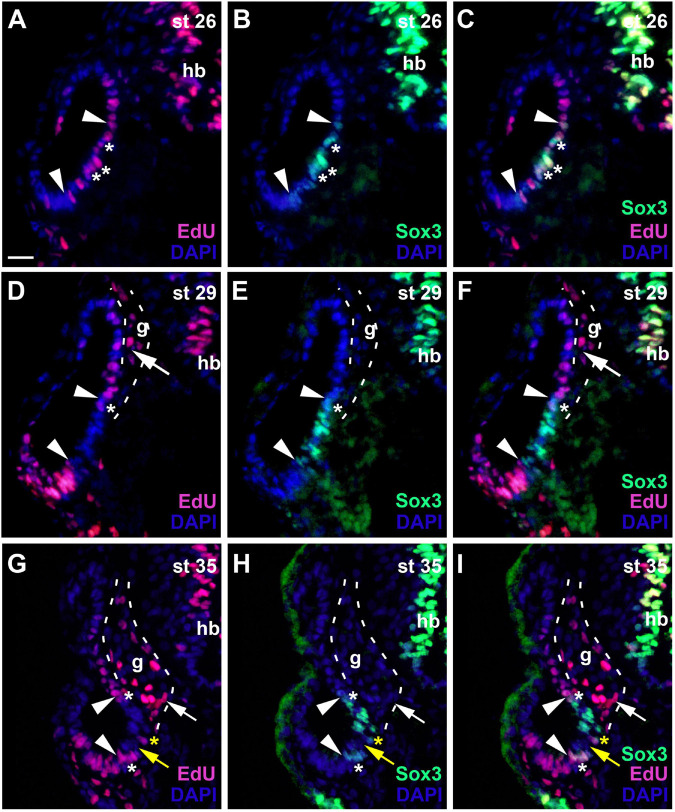
Changing distribution of proliferative and non-proliferative progenitors during development of the otic vesicle. Distribution of proliferative (EdU-positive) cells and Sox3−immunopositive sensorineural progenitors in transverse sections through the center of the left otic vesicle of *Xenopus* embryos from stage 26 to 35 (dorsal to the top, medial to the right). DAPI was used to label nuclei. Different channels of same section shown in **(A–C)**, **(D–F)** and **(G–I)**. g, vestibulocochlear ganglion (outlined with hatched lines); hb, hindbrain. At stage 26 **(A–C)**, Sox3-immunopositive cells are confined to the ventromedial part of the otic vesicle (between arrowheads), located within a broader domain of EdU staining. Most Sox3–immunopositive cells are also labeled with EdU (asterisks indicate double-labeled cells). At stages 29 **(D–F)** and 35 **(G–I)**, most cells in the ventromedial region are immunopositive for Sox3 (region between arrowheads) but are no longer proliferative as indicated by lack of EdU staining. A few cells, which are both EdU- and Sox3-positive remain at the upper and lower border of this domain (white asterisks). From stage 33 on, a region of Sox3-immunonegative nuclei (yellow arrows) separates a dorsal from a ventral domain of Sox3-positive cells within the ventromedial domain as shown here for stage 35. Occasionally single EdU-positive cells, which may also be weakly Sox3-positive as shown here, are found in the region between the dorsal and ventral Sox3 domain (yellow asterisks). Proliferative, EdU-positive cells in the vestibulocochlear ganglion (arrows) are confined to the periphery of the ganglion and do not co-express Sox3. Scale bar in **(A)**: 25 μm (for all panels).

During early invagination of the otic vesicle (stages 20–23) most nuclei are PCNA-positive but PCNA-staining then subsides in nuclei of the lateral wall of the otic vesicle (stage 26) (Supplementary Figures 1A–C). EdU-incorporation at stage 26 confirms that proliferative cells are now scattered throughout the medial wall of the otic vesicle (Figure 5A). Doublestaining for EdU and Sox3 shows that within this proliferative domain, Sox3-immunopositive nuclei are found in a smaller ventromedial area, ranging from mid-dorsal to ventral levels on the medial side of the otic vesicle (Figures 5A–C). Most but not all of these Sox3-immunopositive nuclei are also stained for EdU.

**FIGURE 6:**
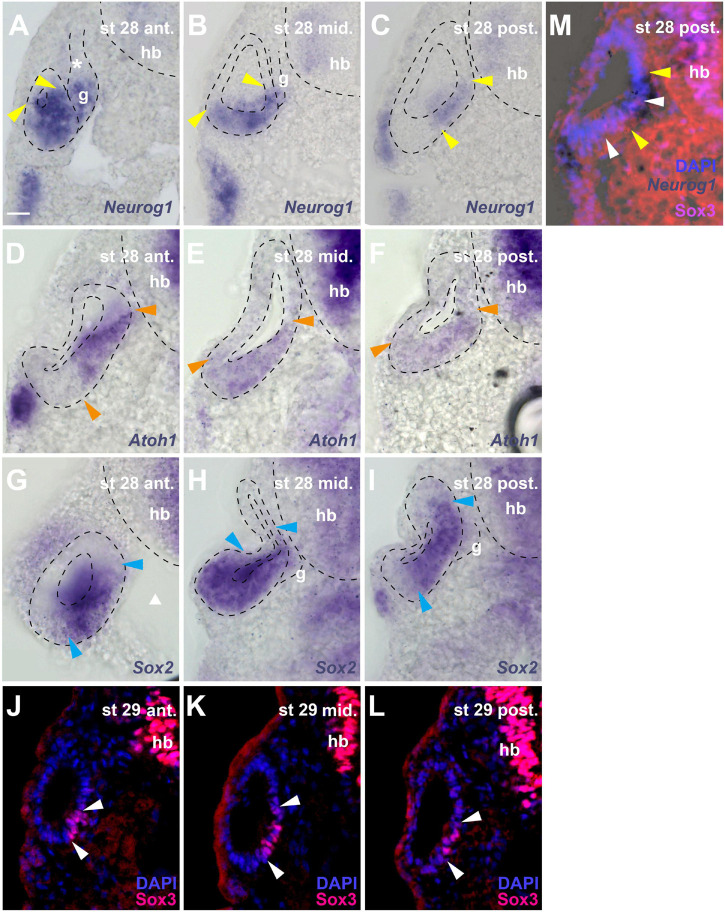
Distribution of neurogenic markers at different levels of the otic vesicle at stage 28/29. Distribution of *Neurog1, Atoh1, Sox2* mRNAs, and Sox3 immunostaining in the left otic vesicle of stage 28 or 29 *Xenopus* embryos showing three approximately equidistant transverse vibratome sections **(A–L)** and a superimposition of *Neurog1* expression with Sox3 immunostaining at a posterior level **(M)**. DAPI was used to label nuclei. Arrowheads indicate extent of region containing cells expressing *Neurog1* (yellow), *Atoh1* (orange), *Sox2* (blue), and Sox3 (white). g, vestibulocochlear ganglion (outlined with hatched lines); hb, hindbrain. Levels: ant.: anterior; mid.: midline; post.: posterior. **(A–C)** Note that at anterior levels, *Neurog1* is expressed throughout the ventral and ventromedial otic epithelium, while it is confined to the central part of the medial otic epithelium further posterior. In the vestibulocochlear ganglion, *Neurog1* is expressed only in the distal part next to the otic epithelium but is absent from the proximal part (asterisk). **(D–F)** *Atoh1* overlaps widely with *Neurog1*, but does not extend as far lateral as the latter in the ventral anterior and more lateral than *Neurog1* in the ventral posterior otic vesicle; it is not expressed in the ganglion. **(G–I)** *Sox2* is expressed very broadly in the central otic vesicle, where its expression extends from midlateral to dorsomedial. Its expression is more restricted anteriorly and posteriorly but always reaches further dorsal in the medial otic epithelium than Sox3. It is also weakly expressed in the distal part of the vestibulocochlear ganglion. **(J–L)** Sox3 immunostaining is confined to the ventromedial otic epithelium reaching its largest dorsal extent in the center of the otic vesicle. **(M)** shows that *Neurog1* expression (between yellow arrowheads in **(M)**) is located slightly more dorsal than Sox3 and is overlapping with the dorsal but not ventral part of the Sox3-domain (between white arrowheads) in the posterior otic vesicle. Scale bar in **(A)**: 25 μm (for all panels).

At subsequent stages, when the otic vesicle has completely invaginated (stages 28–31), EdU and PCNA-staining declines in most nuclei in the ventromedial Sox3-immunopositive domain (Figures 5D–F and Supplementary Figure 1D). The size and position of the Sox3-immunopositive and EdU-negative region varies slightly along the anteroposterior axis and extends furthest dorsally and ventrally at the midline of the inner ear (Figures 6J–L). In the developing vestibulocochlear ganglion (stage 29 onward), which does not show any Sox3-immunostaining, EdU- and PCNA-positive cells are confined to the periphery (in particular on its ventral side) and are absent from its core (Figures 5D–I and Supplementary Figures 1E,F).

Due to the lack of antibodies that recognize Sox2, Neurog1 or Atoh1 in *Xenopus*, we were unable to investigate the precise relation of the Sox3-immunopositive region to the expression domain of the related transcription factor Sox2 or to regions of neuronal or sensory (hair cell) specification as defined by Neurog1 and Atoh1, respectively (Ma et al., 1996, 1998, 2000; Bermingham et al., 1999; Chen et al., 2002; Woods et al., 2004; Nieber et al., 2009; Fritzsch et al., 2010). However, sections through stage 28 or 29 otic vesicles after *in situ* hybridization for *Sox2, Neurog1* or *Atoh1*, show that these regions of expression are partially overlapping (Figure 6).

*Sox2* is expressed much more broadly in the otic vesicle than Sox3 extending to the lateral side and to more dorsomedial areas at the central level of the otic vesicle (Figures 6G–I). In the anterior and posterior otic vesicle, the expression of Sox2 is less extensive but always reaches further dorsal in the medial otic epithelium than Sox3 (Figures 6G–I). Sox2 is also weakly expressed in the ventral part of the vestibulocochlear ganglion immediately adjacent to the otic epithelium, corresponding to the region of strong EdU and PCNA staining (Figures 6H,I).

Based on comparisons between staining patterns, *Neurog1* expression at the anterior and central level of the otic vesicle (Figures 6A,B) possibly extends further ventrolaterally than Sox3 immunostaining (Figures 6J,K) into a region corresponding to the ventral area of cell delamination described above (white arrows in Figures 1E,F,H,I). However, this needs to be confirmed by doublestaining (which we were unable to perform successfully in this region). Conversely, doublestaining with Sox3 reveals that in the posterior otic vesicle the area of *Neurog1* expression extends further dorsal but less far ventral than Sox3 (Figure 6M). This dorsal and Sox3-negative part of the *Neurog1*-positive domain (located dorsal to the bend in the medial wall of the otic vesicle, which is obvious at stages 28–32) approximately corresponds to the area, where the basal lamina is maximally disrupted and most cell delamination occurs suggesting that neuronal progenitors maintain Neurog1 but downregulate Sox3 prior to delamination (compare Figures 1E–I, 6A–C,M). *Neurog1* is also expressed in the ventral part of the vestibulocochlear ganglion immediately adjacent to the otic epithelium, where the strongly proliferating cells reside (Figures 6A,B). *Atoh1* overlaps widely with *Neurog1* in the stage 28 otic epithelium. However, *Atoh1* does not extend as far ventral and lateral as *Neurog1* in the anterior but it extends further ventral and ventrolateral in the posterior otic vesicle than both Neurog1 (Figure 6C) and Sox3 (Figure 6L) and is not expressed in the vestibulocochlear ganglion (Figures 6D–F).

The relatively quiescent region, which contains the Sox3-immunopositive cells and is flanked by proliferative zones dorsally and ventrally is maintained at subsequent stages (stages 35 and 40; Figures 5G–I and Supplementary Figures 1E,F). Only Sox3-immunopositive cells on the dorsal and ventral border of this domain, continue to be double-stained for EdU (Figures 5G–I). From stage 33 on, Sox3-immunostaining in the ventromedial otic epithelium also begins to divide into a dorsal and a ventral domain, which are separated by Sox3-negative nuclei (Figures 5G–I). Occasionally, single PCNA- or EdU-positive nuclei (which may also be weakly Sox3-positive) are found in the middle of the quiescent region, in between the two Sox3 domains (Figures 5G–I and Supplementary Figure 1E). The expression of *Sox2* at stage 35 remains much broader than Sox3 expression and *Sox2*, but not *Sox3*, continues to be expressed in the ventral part of the vestibulocochlear ganglion (compare Supplementary Figures 2D–I). There are some notable changes in the expression of *Neurog1* in the stage 35 otic vesicle compared to stage 28/29. *Neurog1* expression has now declined in the anterior otic vesicle, while its expression has shifted slightly more ventrally in the posterior otic vesicle (Supplementary Figures 2A–C).

Taken together, this indicates that the delaminating neurons and hair cells arise from *Neurog1* and/or *Atoh1* expressing progenitors residing in a common neurosensory area in the ventromedial otic vesicle. Soon after invagination of the otic vesicle is completed (stage 28), this region continues to proliferate at its borders, whereas proliferation declines in most Sox3-immunopositive cells in its central part. The *Neurog1* expression pattern suggests that neurogenesis may take place throughout this domain in the anterior and central otic vesicle and along its dorsal part in the posterior otic vesicle, with the remaining Sox3-positive areas probably contributing to the sensory areas, which also express *Atoh1*. Being more broadly distributed than Sox3, Neurog1 and Atoh1, *Sox2* expression may synergize with Sox3 in defining sensorineural progenitors. However, its expression appears to extend beyond the region of neuronal and sensory progenitors in the otic epithelium (e.g., into the lateral wall) suggesting that Sox2 alone may be insufficient to define a sensorineural progenitor state. Nevertheless, only Sox2 but not Sox3 is expressed in the ventral part of the vestibulocochlear ganglion, where proliferating progenitors reside, suggesting a possible role for Sox2 in neuronal progenitors.

### Differentiation of Sensory Neurons and Early Sensory Areas

**FIGURE 7:**
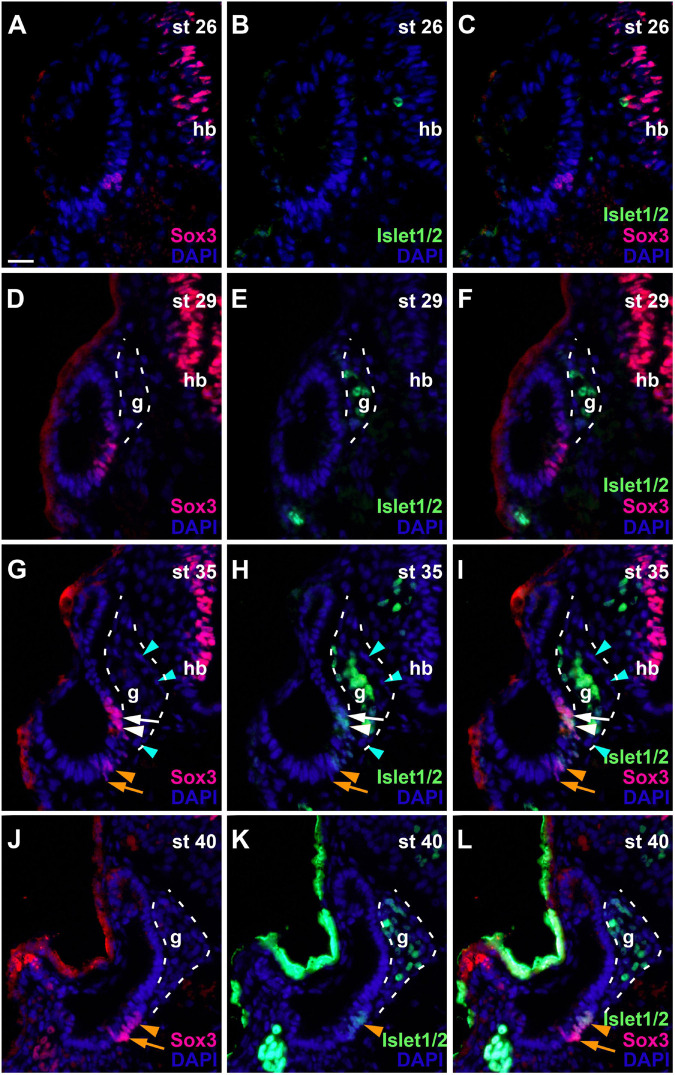
Changing distribution of sensorineural progenitors and differentiating neurons during development of the otic vesicle. Distribution of Sox3-immunopositive sensorineural progenitors in relation to Islet1/2-immunopositive differentiating neurons in transverse sections through the center of the left otic vesicle of *Xenopus* embryos from stage 26 to 40 (dorsal to the top, medial to the right). DAPI was used to label nuclei. Different channels of same section shown in **(A–C)**, **(D–F)**, **(G–I)**, and **(J–L)** (slightly posterior of center). g, vestibulocochlear ganglion (outlined with hatched lines); hb, hindbrain. At stage 26, no Islet1/2 positive cells can be seen. From stage 29 on, strongly Islet1/2 positive cells are evident in the vestibulocochlear ganglion. Note the absence of Islet1/2 staining in the peripheral cells of the ganglion (mint arrowheads). In addition, a subset of Sox3-positive cells in the otic epithelium shows weak Islet1/2 staining (white and orange arrowheads), whereas other Sox3-positive cells do not express Islet1/2 (white and orange arrows) (another more dorsal domain of Sox3 + and weakly Islet1/2 + cells present at stage 40 is only visible in more anterior sections; see Supplementary Figure 3). **(D–F)** Show same section as Figure 6K. Scale bar in **(A)**: 25 μm (for all panels).

To determine the spatiotemporal pattern of neuronal differentiation in the vestibulocochlear ganglion, we used an antibody recognizing Islet1 and Islet2 proteins, which are only expressed in differentiated neurons (Li et al., 2004; Radde-Gallwitz et al., 2004). While no Islet1/2-staining was detected at stage 26 (Figures 7A–C), strongly Islet1/2-immunopositive nuclei became apparent in the vestibulocochlear ganglion from stage 29 on (Figures 7D–F), preceding the outgrowth of the first neurites at stage 32 (Figure 1G). Islet1/2-positive cells were confined to cells in the core of the ganglion, while cells in its periphery do not stain for Islet1/2. This pattern, which persists at stages 35 and 40 (Figures 7G–L), indicates that Islet1/2 is largely or completely absent from proliferating cells, which we have shown to be localized on the outside of the ganglion (Figures 5G–I).

In addition to the ganglion, we also found weaker Islet1/2-staining in the otic epithelium from stage 33 on in some of the Sox3-immunopositive cells. While Islet1/2 is not expressed in the dorsalmost and ventralmost Sox3-immunopositive cells, which we have shown to be proliferative, it is expressed in most of the remaining population (Figures 7G–L and Supplementary Figure 3), presumably defining the developing sensory areas. A division between an upper (more dorsal) and lower (more ventral) part of Sox3- and Islet1/2-doublestained cells separated by immunonegative cells was first observed at stage 33 and becomes more pronounced at stage 35, providing the first indication of a subdivision of the common sensorineural area (Figures 7G–I and Supplementary Figures 3A–I). From stage 35 on, the epithelium on both sides of the dividing line starts to become bilayered with cells with larger nuclei, which remain Sox3- and Islet1/2-positive, positioned basally and Sox3-and Islet1/2-negative cells with smaller nuclei positioned apically (Supplementary Figures 3F–I). This suggests that sensory areas with apically located hair cells and basally located supporting cells start to form. At stage 40, the distinction between the two layers becomes much clearer and the extent of the sensory areas, which will form the utricular macula in the superior (and slightly more anterior) part (Supplementary Figures 3K–N) and the saccular macula in the inferior (and slightly more posterior) part of the otic vesicle, has increased (Supplementary Figures 3O–R). These two macule can also be visualized with acetylated tubulin antibodies, which label the hair bundles of their hair cells (Supplementary Figure 3J). While most of the hair cells in the sensory macule show neither immunostaining for Sox3 nor for Islet1/2, a minority of hair cells is Islet1/2- but not Sox3-positive, suggesting that Sox3 is downregulated before Islet1/2 in hair cell precursors (Supplementary Figures 3K–R).

### Changing Distribution of Eya1 During Development of the Otic Vesicle

To begin to understand the role of Eya1 for otic neurogenesis, we next analyzed the distribution of Eya1 protein in the developing otic vesicle using a *Xenopus*-specific Eya1 peptide antibody raised in guinea-pigs (Ahrens and Schlosser, 2005). This antibody specifically recognizes Eya1 protein and allows to visualize Eya1 immunhistochemically after tyramid signal amplification (Supplementary Figure 4). Eya1 immunostaining completely disappears after addition of the Eya1 peptide against which the antibody was raised confirming the specificity of the signal (Supplementary Figure 4). We found Eya1 immunostaining in all cranial placodes and their derivatives but will here only describe Eya1 distribution in the otic vesicle.

**FIGURE 8:**
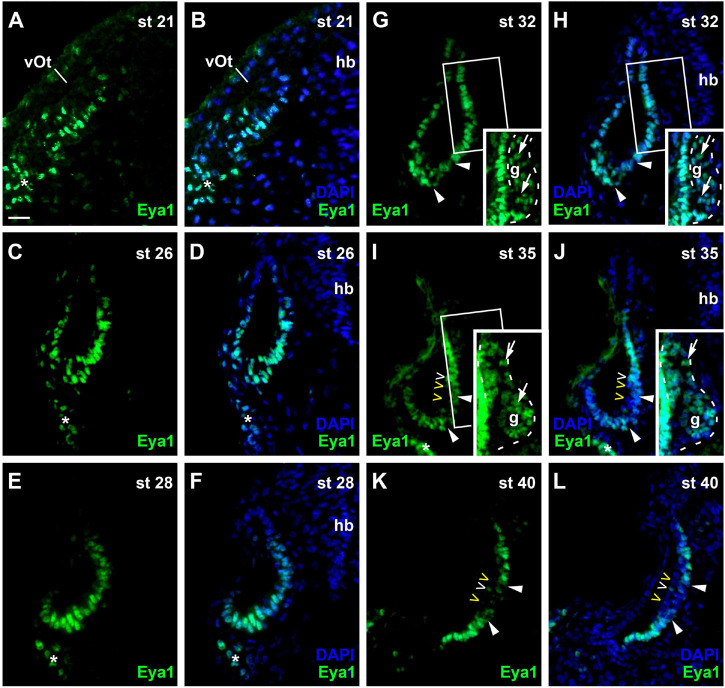
Changing distribution of Eya1 protein during development of the otic vesicle. Distribution of Eya1-immunopositive cells in transverse sections through the center of the left otic vesicle of *Xenopus* embryos from stage 21 to 40 (dorsal to the top, medial to the right). For each stage, Eya1 staining is shown alone (left panel) as well as superimposed onto nuclear DAPI staining (right panel). Insets in **(G–J)** show boxed areas of same or adjacent section at higher magnification and with increased brightness (boxed area not shown in **(J)** for clarity). **(A,B)** At stage 21, Eya1 is expressed in the invaginating otic vesicle and in the adjacent posterior placodal area (asterisk). **(C–F)** At stages 26 **(C,D)** to 28 **(E,F)**, Eya1 immunostaining persists throughout the otic vesicle except for dorsomedial and dorsolateral regions. **(G–J)** From stage 32 **(G,H)** on, additional weak Eya1 staining is found throughout the vestibulocochlear ganglion (arrows in insets, which show boxed regions with increased brightness), while strong Eya1-immunostaining persist in the otic epithelium and in peripheral cells of the ganglion. From stage 32 on, Eya1-immunostaining decreases in a ventromedial region of the otic epithelium (flanked by arrowheads), while it remains high in adjacent regions. From stage 35 **(I,J)** onward, hair cells (open arrowheads) become apparent as a separate layer. While most hair cells do not express Eya1 (yellow open arrowheads), a few hair cells show weak Eya1 staining (white open arrowheads). **(K,L)** At stage 40, Eya1 is still weakly expressed in the vestibulocochlear ganglion (not shown here; see Supplementary Figure 6). In the otic epithelium Eya1 levels are low in the developing sensory areas (between arrowheads) but Eya1 remains strongly expressed in adjacent regions. g, vestibulocochlear ganglion (outlined with hatched lines); hb, hindbrain; vOt, otic vesicle. Scale bar in **(A)**: 25 μm (for all panels).

At stage 21, Eya1 is widely distributed in the posterior placodal area including the invaginating otic vesicle except for its dorsalmost part, where only weak Eya1 immunostaining is observed (Figures 8A,B). At stages 26 and 28, Eya1 remains widely expressed in the otic vesicle but is absent form dorsomedial and dorsolateral regions (Figures 8C–F). As soon as the vestibulocochlear ganglion forms at stage 29, weak Eya1 immunostaining is found throughout the ganglion with stronger Eya1 staining on its periphery, where proliferating cells are localized (Figures 8G–J). At the same time, Eya1 immunostaining begins to weaken in the ventromedial part of the otic epithelium (Figures 8G,H). This region of decreased Eya1 staining in the ventromedial otic epithelium persists in subsequent stages and approximately corresponds to the region of putative sensory areas, where EdU-negative but Sox3- and weakly Islet1/2-positive cells are found at all levels of the developing otic vesicle (Supplementary Figure 5). When the epithelium in this region comprising the putative sensory areas becomes bilayered from stage 35 on, Eya1-staining disappears from most cells in the apical layer but weakly persists in the basal layer suggesting that weak Eya1 expression is maintained in supporting cells but downregulated in differentiating hair cells (Figures 8I–L and Supplementary Figure 6).

### Subcellular Localization of Eya1 in Otic Vesicle and Vestibulocochlear Ganglion

**FIGURE 9:**
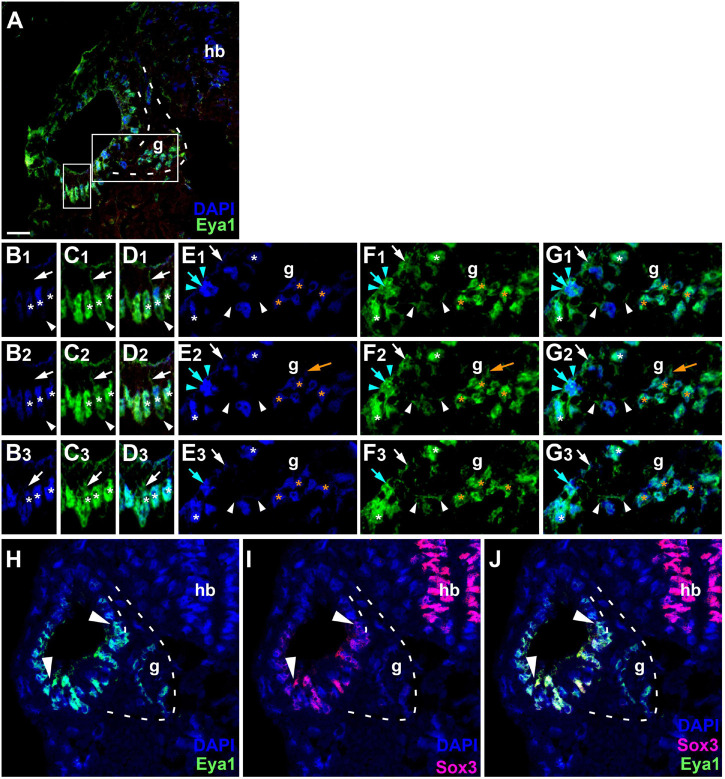
Subcellular localization of Eya1 protein in otic vesicle and vestibulocochlear ganglion at stage 29. **(A–G)** Immunostaining for Eya1 in a transverse section through the center of the left otic vesicle of *Xenopus* embryos at stage 29 analyzed by confocal microscopy (dorsal to the top, medial to the right). DAPI was used to label nuclei. **(A)** Overview showing the same confocal plane as **(B_2_–G_2_)**. **(B–G)** Magnified views of the small **(B–D)** and large **(E–G)** boxed area shown in different channels (columns **B–G**) and in three different confocal planes (rows 1–3; 0.9 μm between adjacent planes). Nuclear staining indicated by asterisks; cytoplasmic staining indicated by arrows. White asterisks show nuclear Eya1 staining in the otic epithelium; orange asterisk indicate Eya1-immunopositive nuclei in periphery of the vestibulocochlear ganglion. White arrows show cytoplasmic staining in the otic epithelium; orange arrows show cytoplasmic Eya1 staining in the ganglion. White arrowheads highlight Eya1-positive protrusions of delaminating or migrating cells. Mint arrowheads indicate cytoplasmic Eya1 staining in a dividing cell of the otic epithelium with the mint arrow indicating the division plane. Note that at this stage cells have begun to delaminate from the otic epithelium to form the vestibulocochlear ganglion. Eya1 shows mostly nuclear but also some cytoplasmic localization in the otic epithelium and vestibulocochlear ganglion. **(H–J)** Immunostaining for Eya1 and Sox3 in a single confocal plane of another transverse section through the center of the left otic vesicle at stage 29. Note that the Eya1 domain includes but extends further dorsally and ventrolaterally than the Sox3 immunopositive domain (between arrowheads). g, vestibulocochlear ganglion (outlined with hatched lines); hb, hindbrain. Scale bar in **(A)**: 25 μm (for **(A,H–J)**).

**FIGURE 10:**
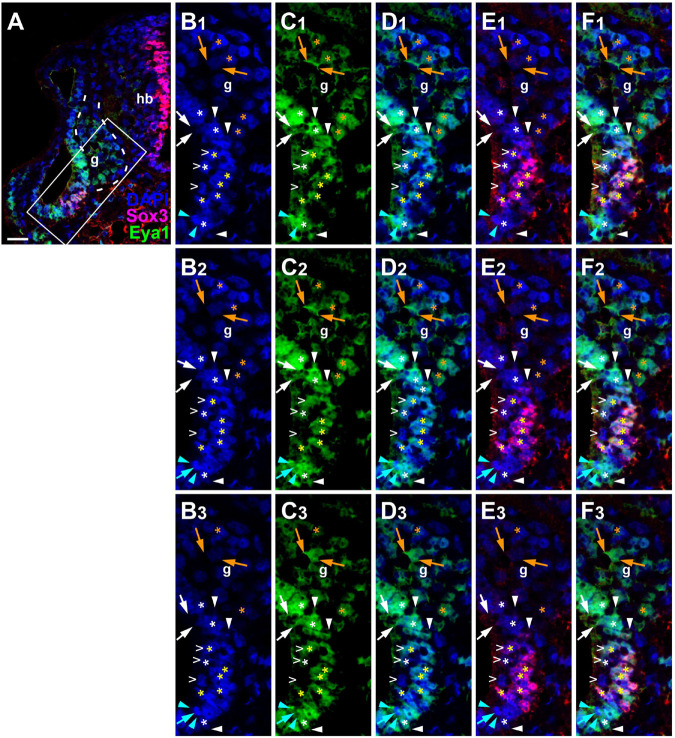
Subcellular localization of Eya1 protein in otic vesicle and vestibulocochlear ganglion at stage 35. Immunostaining for Eya1 and Sox3 in a transverse section through the center of the left otic vesicle of *Xenopus* embryos at stage 35 analyzed by confocal microscopy (dorsal to the top, medial to the right). DAPI was used to label nuclei. **(A)** Overview showing the same confocal plane as **(B_2_–F_2_)**. **(B–F)** Magnified views of the boxed area shown in different channels (columns **B–F**) and in three different confocal planes (rows 1–3; 0.9 μm between adjacent planes). Putative hair cells indicated by white open arrowheads. Nuclear staining indicated by asterisks; cytoplasmic staining indicated by arrows. White asterisks show nuclear Eya1 staining in the otic epithelium; yellow asterisks indicate nuclei that are immunopositive for both Eya1 and Sox3. Orange asterisk indicate Eya1-immunopositive nuclei in the vestibulocochlear ganglion. White arrows show cytoplasmic staining in the otic epithelium; orange arrows show cytoplasmic Eya1 staining in the ganglion. White arrowheads highlight Eya1-positive protrusions of delaminating cells. Mint arrowheads indicate cytoplasmic Eya1 staining in a dividing cell of the otic epithelium with the mint arrow indicating the division plane. Note that Eya1 shows mostly nuclear but also some cytoplasmic localization in the otic epithelium. Sox3-immunopositive nuclei also co-express Eya1, but at lower levels than adjacent cells. A subset of putative hair cells shows weak nuclear Eya1 staining and a subset of the latter also is weakly Sox3-immunopositive. In the vestibulocochlear ganglion, nuclear Eya1 is mostly found in cells located at the periphery (probably corresponding to proliferative cells), while in the center of the ganglion, Eya1 is mostly cytoplasmic. g, vestibulocochlear ganglion (outlined with hatched lines); hb, hindbrain. Scale bar in **(A)**: 25 μm.

Since the subcellular localization of Eya1 has been shown to have important functional implications (Tadjuidje and Hegde, 2013; Rebay, 2016; Roychoudhury and Hegde, 2021), we next used confocal microscopy to study the distribution of Eya1 protein during otic development in more detail. At stages 26 and 29, we find Eya1 predominantly localized to the nuclei in the otic epithelium although some spots of Eya1 are also found in the cytoplasm, where they are concentrated in the apical half of the cells (white arrows in Figure 9 and Supplementary Figure 7). During the main period of neurogenesis as exemplified here by stages 29 and 35, nuclear Eya1 staining is also observed in the periphery of the vestibulocochlear ganglion, while it is predominantly cytoplasmic in the core of the ganglion (Figures 9, 10). In addition, cytoplasmic Eya1 staining is found in cells with basal protrusions, which are probably delaminating from the otic epithelium (white arrowheads in Figures 9, 10). In all of these stages, Eya1 is localized to the cytoplasm (mint arrowheads in Figures 9, 10 and Supplementary Figure 7) and the division plane in dividing cells (mint arrows in Figures 9, 10 and Supplementary Figure 7).

Doublestaining reveals that at stage 29 all Sox3-immunopositive nuclei in the ventromedial part of the otic epithelium are also strongly Eya1-positive (Figures 9H–J). However, nuclear Eya1 is also found dorsal and ventral to the Sox3 domain in the otic epithelium as well as in the peripheral vestibulocochlear ganglion cells, which are Sox3-negative (Figures 9H–J). At stage 35, Eya1 continues to be expressed in Sox3-immunopositive nuclei in the basal part of the bilayered ventromedial otic epithelium (putative supporting cells), but at lower levels than in the Sox3-negative cells in the adjacent otic epithelium or in the peripheral vestibulocochlear ganglion (Figure 10). In contrast, only a subset of cells in the apical part of the ventromedial otic epithelium (putative hair cells) show weak nuclear Eya1 staining and only rarely do the latter also show weak Sox3-immunostaining. Together with our finding that the ventromedial otic epithelium at earlier stages is comprised of Sox3+/Eya1+ cells, this suggests that hair cells arise from Sox3+/Eya1+ progenitors and downregulate first Sox3 and then Eya1. Similarly, the persistence of Eya1 but not of Sox3 in the vestibulocochlear ganglion suggests that Sox3 is downregulated before Eya1 during neurogenesis.

At stage 40, strong nuclear localization of Eya1 persists in cells flanking the developing sensory areas in the superior and inferior part of the otic vesicle dorsally and ventrally, while the nuclei in the basal layer of these sensory area remain only weakly Eya1 positive and only a few nuclei in the apical layer show weak Eya1 staining (Supplementary Figure 8). Interestingly, a similar pattern of Eya1 localization is observed in lateral line neuromasts, where strong nuclear Eya1 staining is confined to a ring of supporting cells, which are highly proliferative as indicated by PCNA staining (Supplementary Figure 8). In contrast, the nuclei of centrally and basally localized supporting cells show weaker Eya1 staining and the centrally and apically localized hair cells are mostly Eya1-negative or retain only very weak Eya1 staining (Supplementary Figure 8).

Taken together, these observations suggest that strong nuclear Eya1 immunostaining is found mainly in proliferating progenitors of the otic epithelium, including (but extending beyond) all progenitors characterized by nuclear Sox3 expression. Strong nuclear expression of Eya1 together with some cytoplasmic expression is then maintained in delaminating neuronal progenitors, which downregulate Sox3. Nuclear Eya1 is subsequently strongly reduced in the differentiating neurons of the vestibulocochlear ganglion, which do, however, maintain Eya1 proteins in the cytoplasm. In the developing sensory areas, weak nuclear Eya1 localization is retained in the relatively quiescent strongly Sox3-positive supporting cells, whereas differentiating hair cells downregulate first Sox3 and then Eya1.

### Role of Eya1 for Otic Neurogenesis

**FIGURE 11:**
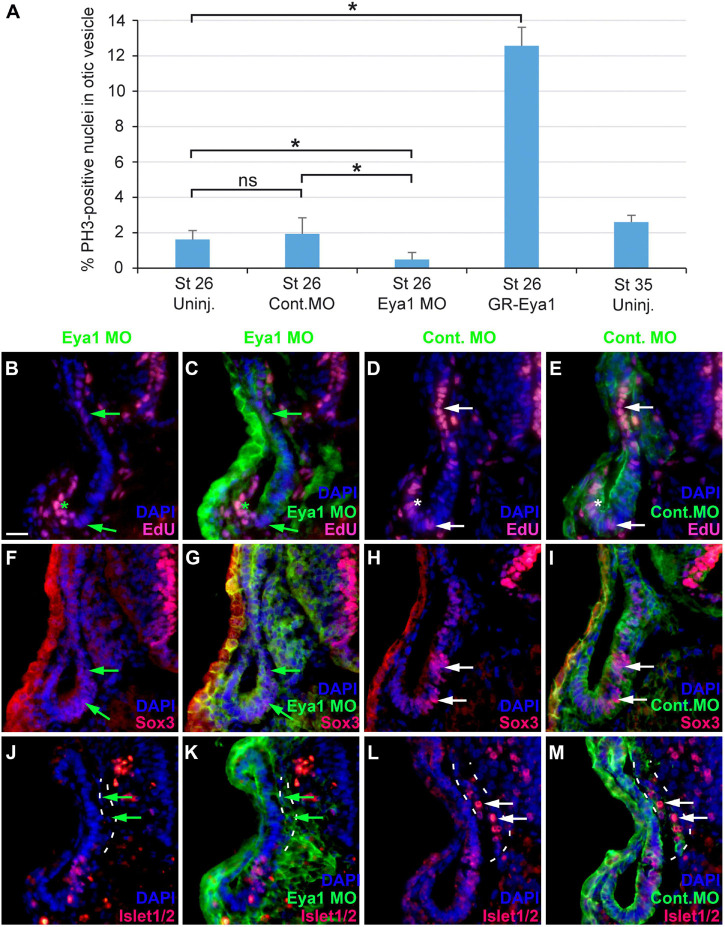
Role of Eya1 for otic neurogenesis in comparisons of embryos injected with Eya1 MOs or Control MOs. **(A)** pH3-immunopositive (mitotic) cells in the stage otic vesicle are unchanged after injection of Control MO (ns, not significant) but are significantly reduced after Eya1 MO injection and significantly increased after GR-Eya1 injection and DEX treatment from stage 16–18 compared to uninjected embryos (Uninj.) (asterisk: *p* < 0.05, *t-*test; *n* = 3 for each condition; standard deviations are indicated). **(B–M)** Changes of EdU-positive proliferative progenitors **(B–E)**, and Sox3- **(F–I)**, and Islet1/2-immunopositive cells **(J–M)** in transverse sections through the central otic vesicle of stage 35 *Xenopus* embryos injected with Eya1 MOs (left two columns; different channels of same section) or control MOs (right two columns; different channels of same section) (dorsal to the top, medial to the right). DAPI was used to label nuclei. Reductions of EdU labeling and Sox3- or Islet1/2-immunoreactive cells in otic vesicle of Eya1 MO injected embryos are indicated by green arrows (compare to white arrows for otic vesicle in Control MO injected embryos). Residual EdU labeling in otic vesicle of Eya1 MO injected embryo is indicated by a green asterisk (compare to white asterisk for otic vesicle in Control MO injected embryo). Vestibulocochlear ganglion outlined with hatched lines in **(J–M)**. Scale bar in **(B)**: 25 μm (for **(B–M)**).

To further characterize the role of Eya1 for otic neurogenesis, we analyzed the consequences of Eya1 knockdown or overexpression for cell proliferation, and the distribution of progenitors and differentiating neurons in the otic vesicle. For Eya1 knockdown we injected a combination of two Eya1 morpholinos (MO), which were previously shown to effectively and specifically abolish Eya1 translation (Schlosser et al., 2008). Co-injection of Eya1 MOs but not of an unspecific control MO drastically reduced or abolished Eya1 immunostaining in the otic vesicle (Supplementary Figure 9). Eya1 MO injection also significantly reduced the percentage of pH3-immunopositive cells in the otic vesicle at stage 26 and led to a decrease in EdU-staining (Figures 11A–E; Supplementary Figures 10A,B; and Supplementary Table 2), indicating that proliferation of otic progenitors is reduced. The number of Sox3-immunopositive cells in the ventromedial otic epithelium and of Islet1/2-immunopositive cells in the ganglion and in the otic epithelium was also reduced (Figures 11F–M and Supplementary Figures 10C–H). None of these changes were observed after injections of Control MO. This indicates that Eya1 is required for proliferation, formation of Sox3-positive progenitors and the differentiation of sensory neurons in the otic epithelium.

**FIGURE 12:**
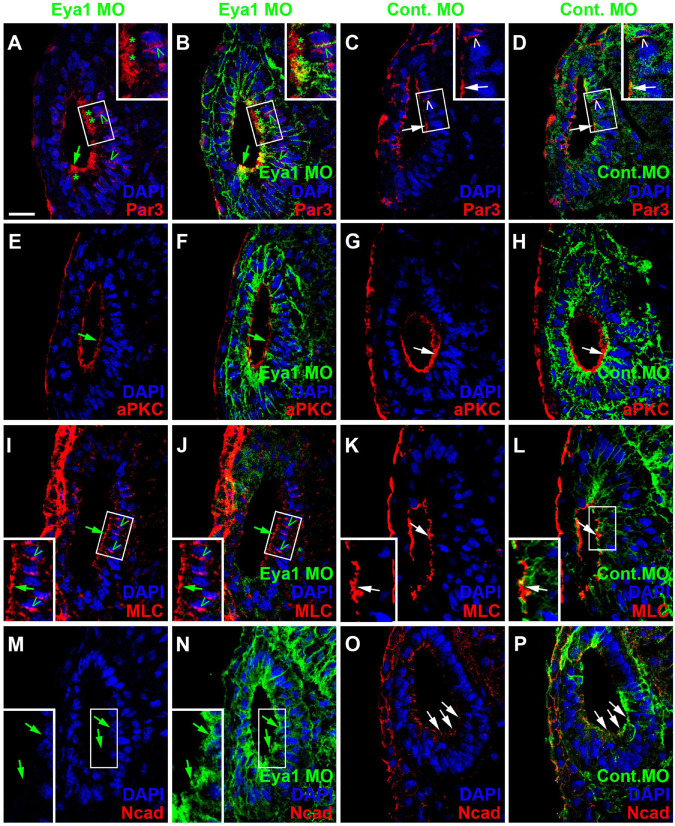
Role of Eya1 for otic cell polarity in comparisons of embryos injected with Eya1 MOs or Control MOs. Changes of Par3- **(A–D)**, aPKC- **(E–H)**, MLC- **(I–L)**, and N-Cadherin **(M–P)** immunostaining in transverse sections through the central otic vesicle of stage 26 *Xenopus* embryos injected with Eya1 MOs (left two columns; different channels of same section) or control MOs (right two columns; different channels of same section) (dorsal to the top, medial to the right). DAPI was used to label nuclei. Embryos injected with Control MOs show a normal pattern of protein distribution (see Figure 2). Boxed areas are shown at higher magnification in insets. Protein distribution in the apical and apicolateral membrane (arrows), cytoplasm (asterisks) and between nuclei and membrane (open arrowheads) are indicated. Green symbols indicate protein distribution in Eya1 MO injected embryos and white symbols in Control MO injected embryos. Note that Par3, aPKC, and MLC remain apically localized after Eya1 MO injection, although apical protein levels of Par3 and aPKC are often reduced compared to embryos injected with Control MO. Apicolateral staining of N-cadherin is completely abolished after Eya1 MO injections but not affected after injection with Control MOs. Increasing cytoplasmic and perinuclear distribution of Par3 and MLC is observed in embryos injected with Eya1 MO as compared to Control MO injected embryos. Scale bar in **(A)**: 25 μm (for all panels).

Because changes in the distribution of cell polarity proteins accompany and possibly help to regulate the transition between epithelial progenitors and delaminating neurons in the otic vesicle (see above), we next analyzed whether knockdown of Eya1 also affected the distribution of cell polarity proteins. While injections of unspecific Control MOs had no effects, Eya1 MO injection resulted in some reduction or redistribution of cell polarity proteins. Although Par3, aPKC and MLC remain apically localized after Eya1 MO injection, apical protein levels of Par3 and aPKC are reduced and cytoplasmic and/or perinuclear distribution of Par3 and MLC is increased in comparison to the uninjected side of the same embryo or to embryos injected with Control MO (Figures 12A–L and Supplementary Figures 11A–F). Most strikingly, apicolateral staining for N-Cadherin is completely abolished in the otic epithelium after Eya1 MO but not after Control MO injection (Figures 12M–P and Supplementary Figures 11G,H). This suggests that Eya1 is required to maintain proper apicobasal polarity and apicolateral N-cadherin in the otic epithelium while its downregulation may promote N-cadherin depletion and possibly delamination.

We next overexpressed Eya1 or the transcription factor Six1, with which Eya1 is thought to cooperate in the regulation of neurogenesis, by injecting mRNAs for the inducible constructs GR-Eya1 or GR-Six1 and inducing nuclear translocation by addition of dexamethasone from neural fold stages (stage 16–18) on. This led to increased EdU incorporation in the otic vesicle after both Eya1 and Six1 overexpression and a significant increase of pH3-staining (only determined for GR-Eya1) suggesting increased cell proliferation in the otic vesicle (Figure 11A; Supplementary Figures 12A–F; and Supplementary Table 2). The ventromedial domain of Sox3-immunopositive cells was also increased after Six1 but not after Eya1 overexpression (Supplementary Figures 12G–L). Islet1/2-immunostained cells in the vestibulocochlear ganglion were slightly decreased in some embryos after either Eya1 or Six1 overexpression (Supplementary Figures 12M–R), but were slightly increased in one embryo injected with GR-Six1 (not shown). No obvious changes in the distribution of cell polarity proteins (Par3, aPKC, MLC) or N-Cadherin were observed after Eya1 overexpression (Six1 overexpression was not analyzed) (Supplementary Figure 13).

## Discussion

### Development of the Early Otic Vesicle in *Xenopus*

Our study provides the first detailed description of otic neurogenesis in *Xenopus laevis*. We observed delamination of neurons from the sensorineural area in the medial otic vesicle in *Xenopus* between stage 26/27 and stage 39. During these stages, cells are seen to form basal protrusions, which mirror deformations and disruption in the basal lamina similar to what has been observed in other vertebrates (Meier, 1978; Carney and Silver, 1983; Whitehead and Morest, 1985a,b; Hemond and Morest, 1991) and suggesting that the basal lamina gets displaced and dissolved by cellular protrusions. Based on Islet1/2 expression and acetylated tubulin staining, the first neurons of the vestibulocochlear ganglion differentiate and send out their first neurites at stage 29–32. The first hair cells in the developing sensory areas differentiate from the sensorineural area at about the same time (stage 31; Quick and Serrano, 2005), followed by the separation into multiple sensory areas from stage 33 on. Therefore, in *Xenopus* otic neurogenesis proceeds in parallel with the differentiation of the first otic hair cells. This time course resembles zebrafish, but is different from amniotes, where most neurons delaminate before the onset of hair cell differentiation (Table 2; Haddon and Lewis, 1996; Adam et al., 1998; Pujades et al., 2006; Raft et al., 2007).

**TABLE 2:** Comparison of inner ear development in different vertebrates.

Event	Pre-placodal region (PPR)	Otic placode	Otic cup	Otic vesicle	Migration of neuroblasts into vestibulocochlear ganglion	Differentiation of ganglion cells/first neurites	Sensory areas begin to separate	First hair cells differentiate	Development of otolith organs and semicircular canals completed
***Xenopus***	**NF 14** (Schlosser and Ahrens, 2004)	**NF 21** (Schlosser and Northcutt, 2000)	**NF 22/23** (Schlosser and Northcutt, 2000)	**NF 28** (Schlosser and Northcutt, 2000)	**NF 27–39** This study	**NF 29/32** This study	**NF 33** This study	**NF 31** (Quick and Serrano, 2005)	**NF 47** (Quick and Serrano, 2005)
**Zebrafish**	**10 hpf (1 som)** (Akimenko et al., 1994; Sahly et al., 1999)	**14 hpf (10 som)** (Haddon and Lewis, 1996)	−	**19.5 hpf (21 som)** (Haddon and Lewis, 1996)	**17 to 42 hpf** (Haddon and Lewis, 1996; Vemaraju et al., 2012)	**24/30 hpf** (Haddon and Lewis, 1996)	**24 hpf** (Haddon and Lewis, 1996)	**24 hpf** (Haddon and Lewis, 1996)	**72 hpf** (Haddon and Lewis, 1996)
**Chick**	**HH 7 (23–26 h; 1 som)** (Esteve and Bovolenta, 1999; McLarren et al., 2003)	**HH 10 (33–38 h; 10 som)** (Hilfer et al., 1989)	**HH 12 (45–49 h; 16 som)** (Hilfer et al., 1989; Wu and Oh, 1996)	**HH 17 (52–64 h)**Wu and Oh, 1996)	**HH 16–28 (51 h–6 d)** (D’Amico-Martel and Noden, 1983; Hemond and Morest, 1991; Bell et al., 2008	**HH 26 (5 d)** (Bartolami et al., 1991)	**HH 19–24 (3–4.5 d)** (Oh et al., 1996; Wu and Oh, 1996)	**HH 26 (5 d)** (Bartolami et al., 1991; Oh et al., 1996)	**HH 30 (6.5–7 d)** (Bissonnette and Fekete, 1996)
**Mouse**	**E8** (Sato et al., 2010)	**E8.5** (Anniko and Wikstrom, 1984)	**E9** (Anniko and Wikstrom, 1984)	**E9.5** (Anniko and Wikstrom, 1984)	**E9-E11.5** (Carney and Silver, 1983; Wikstrom and Anniko, 1987; Ma et al., 1998; Raft et al., 2004)	**E11.5** (Carney and Silver, 1983)	**E11.5** (Morsli et al., 1998; Raft et al., 2007)	**E11.5** (Shailam et al., 1999)	**E13 to E17** (Morsli et al., 1998)

Our findings at stage 26 show that toward the end of invagination the *Xenopus* otic epithelium is a typical pseudostratified epithelium with apical mitoses and with localization of Par3, aPKC (see also Jung et al., 2011), MLC and N-cadherin to the apical and/or apicolateral membrane (Knoblich, 2010; Chen and Zhang, 2013; Vorhagen and Niessen, 2014; Miyamoto et al., 2015). Surprisingly, we also found some localization of Par3, aPKC and MLC to the periphery of nuclei and to the adjacent cell membrane. This raises the possibility that these proteins may play a role in the mechanism driving interkinetic nuclear migration (IKNM). IKNM is thought to be mediated by actin and myosin in short pseudostratified epithelia such as the otic epithelium, while it involves microtubule-dependent processes in pseudostratified epithelia with longer cells (Norden et al., 2009; Tsai et al., 2010; Kosodo et al., 2011; Leung et al., 2011; Strzyz et al., 2015; Norden, 2017). Cell polarity proteins have so far not been implicated in this process, but our data suggest that they may play a role, for example in anchoring cytoskeletal proteins to both the nucleus and the cell membrane or in regulating their dynamics. This been shown in other contexts (Etienne-Manneville and Hall, 2003; Suzuki and Ohno, 2006; Hapak et al., 2018) but remains to be tested experimentally for the inner ear.

We also report here for the first time that the distribution of cell polarity proteins in the otic vesicle changes significantly, when cells begin to delaminate. Both Par3 and aPKC are then becoming localized to basal protrusions as well as to cell membranes of vestibulocochlear ganglion cells including their axon and dendrite forming processes. At the same time, Par3 and MLC become depleted from the apical membrane of the otic vesicle. This raises the possibility that the re-distribution of cell polarity proteins may play some causal role in regulating the transition from epithelial cells to delaminating neurons in the otic epithelium. Since Par3 and aPKC have been implicated in neuronal cell migration and neurite outgrowth in the vertebrate nervous system (Shi et al., 2003; Du et al., 2010; Solecki, 2012; Chen and Zhang, 2013; Moore et al., 2013; Ramahi and Solecki, 2014; Hapak et al., 2018), our findings further suggest that they may play similar roles for the delaminating vestibulocochlear ganglion cells. However, this needs to be investigated in further studies.

### Neurogenesis in the Otic Vesicle

**FIGURE 13:**
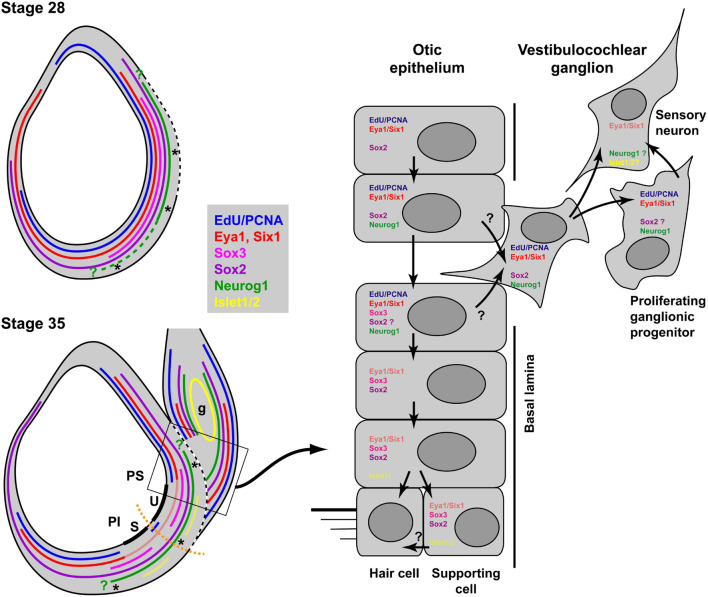
Neurogenesis and sensory area formation in the otic vesicle of *Xenopus laevis*. Schematic diagrams of central sections through otic vesicles are shown with proliferation zones (EdU/PCNA) and approximate extent of marker expression domains in the otic vesicle and the vestibulocochlear ganglion (g) indicated by colored lines. Faint red and yellow colors indicate domains of weak expression of Eya1 and Islet1/2, respectively. Data from previous publications suggest that Six1 is expressed in similar domains to Eya1 (Pandur and Moody, 2000; David et al., 2001; Schlosser and Ahrens, 2004). All other data are based on the current study. Green question marks indicate that the precise position of the expression boundaries for Neurog1 are not known. The hatched part of the line for Neurog1 indicates that its expression extends more ventrally in the anterior otic vesicle at stage 28. The broken white line indicates the extent of the breach in the basal lamina. Thick black lines indicate the developing sensory areas (maculae) of the saccule (S) in the pars inferior (PI) and of the utricle (U) in the pars superior (PS) of the otic vesicle. Asterisks indicate regions of neuronal delamination. These extend broadly throughout the ventromedial part of the otic vesicle at early stages but probably become restricted to the ventral and dorsal borders of sensory areas at stage 35. Black arrows in the right panel indicate putative cell state transitions. See text for details.

Double immunostaining for EdU and Sox3 or Islet1/2 and Sox3 allowed us to document the progression of neuronal differentiation and sensory area formation from a common sensorineural area in the *Xenopus* otic vesicle as summarized in Figure 13. When neurogenesis starts in *Xenopus* around stages 26–28, Sox3-immunopositive cells are found in proliferating cells throughout the region where the basal lamina is interrupted and/or neuroblasts are delaminating. While Sox3 overlaps with *Neurog1* expression in the region where neuroblasts are delaminating, cells in the dorsalmost part of the *Neurog1* expression domain, do not express Sox3 and the latter is also completely absent from the vestibulocochlear ganglion. This suggests, that neuronal progenitors maintain Neurog1 but downregulate Sox3 prior to delamination (Figure 13). This is similar to zebrafish and amniotes, where downregulation of SoxB1 factors has been shown to be required for the differentiation of sensory neurons (Dabdoub et al., 2008; Millimaki et al., 2010; Evsen et al., 2013), while Neurog1 is required for initiating differentiation of otic sensory neurons (Ma et al., 1996, 1998, 2000; Andermann et al., 2002).

Unfortunately, we were not successful in our attempts to also study the distribution of Sox2 in the otic vesicle immunohistochemically. However we show here that *Sox2* mRNA is expressed more broadly during the period of otic neurogenesis and extends further dorsally in the medial wall than Sox3 (see also David et al., 2001; Schlosser and Ahrens, 2004; Park and Saint-Jeannet, 2010). We further show that *Sox2* is also expressed in the proliferative peripheral ganglion cells. This pattern suggests that Sox2 rather than Sox3 helps to define neuronal progenitors in the vestibulocochlear ganglion, similar to what has been reported for mammals, but different from chick and zebrafish (Kiernan et al., 2005; Neves et al., 2007; Millimaki et al., 2010; Evsen et al., 2013; Steevens et al., 2017; Gou et al., 2018). In the otic epithelium, *Sox2* appears to be co-expressed with Sox3 in regions where neurons delaminate. This is compatible with a role of either Sox2 or Sox3 or both in regulating a neuronal progenitor state, although the broad distribution of Sox2 in the otic vesicle, clearly extending beyond regions of neuronal delamination, suggests that Sox2 alone is not sufficient to specify neuronal progenitors in the otic epithelium.

Proneural proteins such as Neurog1 are known to act upstream of and in cooperation with other transcription factors to specify sensory and neuronal cell types, including LIM-type (e.g., Islet1) and POU-type homeodomain proteins (Alsina, 2020; Lee and Pfaff, 2003; Ma et al., 2008; Lee et al., 2012). In accordance with this, we find strong Islet1/2 expression restricted to the non-proliferating central cells of the vestibulocochlear ganglion in *Xenopus* indicating that Islet1/2 is upregulated in differentiating neurons. Similarly, Islet1/2 has been previously shown to be strongly expressed in the differentiating neurons of the chicken inner ear (Adam et al., 1998; Camarero et al., 2001; Begbie et al., 2002; Li et al., 2004; Deng et al., 2014; Durruthy-Durruthy et al., 2014), and has been implicated in their specification and maintenance (Li et al., 2004).

### Development of Sensory Areas

The domain of Sox3 immunostaining in the medial wall of the otic vesicle probably extends more ventral than *Neurog1* expression into an area which expresses only *Atoh1* but not *Neurog1*, and Sox3 is maintained in this ventromedial domain (mostly in non-proliferative cells including supporting cells) even at stage 40, when the delamination of neuroblasts has been completed. From stage 33 on, this ventromedial region becomes thickened and bilayered indicating the formation of the first sensory area, which begins to separate into a dorsal and ventral part from stage 33 on. These will subsequently give rise to the utricular and saccular macula, respectively, and probably contribute to the formation of additional sensory areas at later stages (Quick and Serrano, 2005). Like in chick embryos, some of the putative supporting cells in the more basal layer of the sensory areas (which express Sox3 in *Xenopus* but Sox2 in the chick), are also weakly immunopositive for Islet1/2 (Neves et al., 2007). The expression of Islet1/2, a transcription factor with a central role in specifying sensory neuronal identity, in both the sensory and neuronal cell lineage lends some support to the proposed evolutionary sister cell relationship between the sensory cells and sensory neurons derived from the otic vesicle (Fritzsch et al., 2002).

The distribution of Sox3 in regions of the otic epithelium, from which neurons will subsequently delaminate, as well as in the developing sensory areas suggests that Sox3 marks a common neurosensory area in the *Xenopus* otic vesicle, possibly in conjunction Sox2 which is expressed in partly overlapping domains but extends more dorsally. A similar neurosensory area containing progenitors for the sensory and supporting cells of utricular and saccular macula and for the sensory neurons of the vestibulocochlear ganglion has been described in zebrafish, chick and mammals (Adam et al., 1998; Ma et al., 1998; Andermann et al., 2002; Alsina et al., 2004; Satoh and Fekete, 2005; Pujades et al., 2006; Millimaki et al., 2007, 2010; Raft et al., 2007; Sapede et al., 2012). However, while Sox3 precedes Sox2 expression and promotes neural differentiation in chick and zebrafish embryos, only Sox2 is maintained in their developing sensory areas and promotes sensory differentiation (Neves et al., 2007; Abelló et al., 2010; Evsen et al., 2013; Gou et al., 2018). In mammals, the neurosensory area expresses only Sox2, which is required for both neuronal and sensory differentiation (Kiernan et al., 2005; Puligilla et al., 2010; Steevens et al., 2017). Since persistent expression of Sox2 and/or Sox3 blocks neuronal or sensory differentiation, these transcription must be downregulated before sensory neurons or hair cells differentiate in all vertebrates (Dabdoub et al., 2008; Evsen et al., 2013; Puligilla and Kelley, 2017). Taken together, these comparisons suggest that either Sox3 or Sox2 or both play a central role for the formation of sensory and neuronal progenitors in the developing inner ear of all vertebrates but that their respective role for sensori- or neurogenesis has changed during vertebrate evolution with Sox3 presumably adopting a more central role for sensorigenesis in *Xenopus* than in other vertebrates.

### Distribution of Eya1 in the Developing Otic Vesicle

It has previously been shown that binding of Eya1 protein to the Six1 transcription factor results in nuclear translocation of Eya1, where Eya1 then acts as a transcriptional coactivator of Six1 (Ohto et al., 1999; Zhang et al., 2004). Together the Six1-Eya1 protein complex then promotes both the proliferation of sensory and neuronal progenitors and the subsequent differentiation of neurons and sensory cells in the otic placode and vesicle and in derivatives of other cranial placodes (Laclef et al., 2003; Li et al., 2003, 2020; Zheng et al., 2003; Schlosser et al., 2008; Zou et al., 2006, 2008; Ahmed et al., 2012a,b; Riddiford and Schlosser, 2016, 2017). Previous studies demonstrated that this occurs in a dosage-dependent manner with high levels of Eya1/Six1 promoting a progenitor state and low levels promoting differentiation (Schlosser et al., 2008; Zou et al., 2008; Riddiford and Schlosser, 2017). Using a specific Eya1 antibody in combination with EdU-incorporation and Sox3- and Islet1/2-immunostaining, we now show that the distribution of Eya1 protein in progenitors and differentiating neurons of the developing inner ear is consistent with the previously proposed dosage-dependent action of Eya1.

High levels of nuclear Eya1 are found only in proliferating (EdU+, Islet1/2−) progenitor cells of the otic epithelium flanking the neurosensory area (including the Sox3+ cells at its dorsal and ventral border) and in the proliferating (EdU+, Islet1/2−) peripheral cells of the vestibulocochlear ganglion (Figure 13). In contrast, lower levels of nuclear Eya1 are found in the relatively quiescent (EdU−) supporting cells of the sensory areas, which co-express Sox3 and low levels of Islet1/2 protein, while nuclear Eya1 staining disappears as soon as neurons and hair cells differentiate. Levels of nuclear Eya1, thus, decline along the trajectory from progenitors to differentiating cells in both the neuronal and sensory lineages. In addition to nuclear staining, we find Eya1 in the cytoplasm, in particular in delaminating neuroblasts and the differentiating (Islet1/2+) neurons of the vestibulocochlear ganglion.

### Role of Eya1 in Early Otic Development

Our loss of function experiments suggest that Eya1 is indeed essential for maintaining proliferation and Sox3-immunostaining in the developing neurosensory area. Eya1 most likely acts as a transcriptional coactivator of Six1 in this context, since Six1 and Eya1 have previously been shown to cooperate in the direct activation of Sox2 and Sox3 and are jointly required for Sox2 expression in the neurosensory area of the mouse (Schlosser et al., 2008; Riddiford and Schlosser, 2016, 2017; Xu et al., 2021). Similarly, the reduction of Islet1/2-positive sensory neurons that we observe here after Eya1 knockdown has been shown to be mirrored by a similar reduction after Six1 knockdown in a previous study, which also identified Islet2 as a putatively direct transcriptional target of Six1 and Eya1 (Riddiford and Schlosser, 2016). Taken together this suggests that Eya1 and Six1 directly transcriptionally activate genes promoting progenitor status (possibly at higher doses) as well as those promoting neuronal differentiation (possibly at lower doses).

We previously reported that *Islet2* as well as *Neurog1/2* expression in cranial placodes may be either increased or decreased after overexpression of Eya1 or Six1 (Schlosser et al., 2008; Riddiford and Schlosser, 2016, 2017). Here we show that Islet1/2 immunostaining in the vestibulocochlear ganglion may likewise be decreased or increased after Six1 overexpression although we only observed reductions after Eya1 overexpression (based on our previous findings, we expect to see occasional increases also after Eya1 overexpression if more embryos are analyzed). These seemingly paradoxical findings may result from the dosage dependent effects of Eya1/Six1 on progenitor proliferation and neuronal or sensory differentiation. In promoting proliferation and upregulating Sox2/3 in the neurosensory area, high levels of Eya1/Six1 expand the pool of neuronal progenitors, which are, however, blocked from differentiation by high levels of Sox2/3. Maintenance of high levels of Eya1/Six1 should therefore repress neuronal differentiation resulting in reduced numbers of Islet1/2 cells in the ganglion. Should, however, levels of Eya1 and/or Six1 subsequently decline sufficiently in the otic vesicle, the expanded progenitor pool may contribute to increased numbers of Islet1/2 cells. The precise levels of Eya1 and/or Six1 and their dynamics after overexpression may, therefore determine, whether neuronal differentiation is increased or decreased.

Our findings indicate that in addition to its effects on proliferation and neuronal differentiation, knockdown of Eya1 also affects the distribution of cell polarity proteins and N-cadherin in the developing otic vesicle (possibly with indirect effects on the balance between progenitors and differentiating cells): Apical protein levels of Par3 and aPKC were reduced and cytoplasmic localization of Par3 and MLC were increased, while N-cadherin disappeared completely from the apicolateral membrane.

The downregulation of N-cadherin after Eya1 knockdown is particularly noteworthy since it suggests that Eya1 may be required to maintain epithelial integrity of the pseudostratified otic epithelium and that the reduction of Eya1 levels may promote delamination. We have shown here that during normal development of the otic vesicle apicolateral N-cadherin staining is relatively weak in areas of the otic vesicle, where neuroblast delamination occurs. This is reminiscent of the downregulation of N-cadherin in regions of neural crest delamination (Akitaya and Bronner-Fraser, 1992; Nakagawa and Takeichi, 1998; Davidson and Keller, 1999; Dady et al., 2012; Rogers et al., 2013). Such downregulation of N-cadherin has been previously shown to be required for initial delamination of the neural crest. Whereas overexpression of N-cadherin prevents neural crest delamination, blocking N-cadherin results in precocious migration (Bronner-Fraser et al., 1992; Nakagawa and Takeichi, 1998; Shoval et al., 2007; Taneyhill and Schiffmacher, 2017). If N-cadherin plays a similar role in the otic vesicle, one role of Eya1 maybe to promote N-cadherin expression in the otic epithelium thereby maintaining an epithelial phenotype, while downregulation of Eya1 may lead to reduction of N-cadherin, thereby permitting delamination. Under the assumption that high levels of Eya1 prevent delamination, the number of differentiating neurons in the vestibulocochlear ganglion should increase after Eya1 knockdown. However, this will be difficult to verify *in vivo* since Eya1 knockdown at the same time reduces progenitor proliferation, thereby reducing the rate with which new cells are produced. Therefore, the proposed role of Eya1 in delamination needs to be confirmed in further studies *in vitro*.

We currently do not know whether Eya1 affects the distribution of cell polarity proteins and N-cadherin via transcriptional regulation of the genes encoding these proteins or by direct protein-protein interactions in the cytoplasm, where the phosphatase activity of Eya1 may play a role in dephosphorylation of protein interaction partners, which may in turn affect their subcellular localization (Rebay, 2016; Merk et al., 2020; Roychoudhury and Hegde, 2021). The latter scenario receives some support from our observation that cytoplasmic Eya1 protein is present in basal protrusions of delaminating cells and in the developing neurites of ganglion cells, where it may interact with Par3 and aPKC. Dephosphorylation of aPKC by Eya1, has already been shown to inactivate the cell polarity complex in cerebellar neurons leading to changes in microtubule orientation and distribution of Numb protein (Merk et al., 2020), but whether similar processes occur in the otic epithelium is currently unclear.

Finally, our findings that Eya1 is required for the proper distribution and stabilization of cell polarity proteins (whether by transcriptional regulation or direct protein-protein interactions) as well as for the maintenance of proliferative progenitors in the otic vesicle and for neuronal differentiation raise the possibility that there may be a causal link between the effects of Eya1 on cell polarity and on neurogenesis in the developing inner ear. In the cerebellum, dephosphorylation of aPKC by Eya1 affects the balance between symmetric and asymmetric cell divisions (with Eya1 promoting symmetric divisions resulting in two proliferative progenitors over asymmetric divisions giving rise to one progenitor and one differentiating cell) and, therefore, helps to regulate the transition between proliferating progenitors and differentiating neurons (Merk et al., 2020). Similarly, by modulating apicobasal cell polarity changing levels of Eya1 may affect the proportion of symmetric versus asymmetric cell divisions during otic development, which in turn may affect the balance between proliferating progenitors and differentiating neurons (Zheng et al., 2003; Zou et al., 2006; Schlosser et al., 2008; Schlosser, 2010). Further studies are needed to test this hypothesis.

## Data Availability Statement

The original contributions presented in the study are included in the article/Supplementary Material, further inquiries can be directed to the corresponding author/s.

## Ethics Statement

The animal study was reviewed and approved by Animal Care Research Ethics Committee (ACREC), NUI Galway, University Rd., Galway, Ireland, Acrec@nuigalway.ie.

## Author Contributions

SA conducted the experiments and contributed to data analysis and writing of the paper. GS designed the study and contributed to data analysis and writing of the paper. Both authors contributed to the article and approved the submitted version.

## Conflict of Interest

The authors declare that the research was conducted in the absence of any commercial or financial relationships that could be construed as a potential conflict of interest.

## Publisher’s Note

All claims expressed in this article are solely those of the authors and do not necessarily represent those of their affiliated organizations, or those of the publisher, the editors and the reviewers. Any product that may be evaluated in this article, or claim that may be made by its manufacturer, is not guaranteed or endorsed by the publisher.
