# Supplementary material for: Otic Neurogenesis in Xenopus laevis: Proliferation, Differentiation, and the Role of Eya1
Source: Front Neuroanat. 2021 Sep 20;15:722374. doi: 10.3389/fnana.2021.722374 (PMC8488300; doi:10.3389/fnana.2021.722374)
Supplement: Supplementary file 1 [file Data_Sheet_1.docx]

**Otic neurogenesis in *Xenopus laevis*:**

**Proliferation, differentiation, and the role of Eya1**

**Supplemental material**

**Supplemental Figures**

**
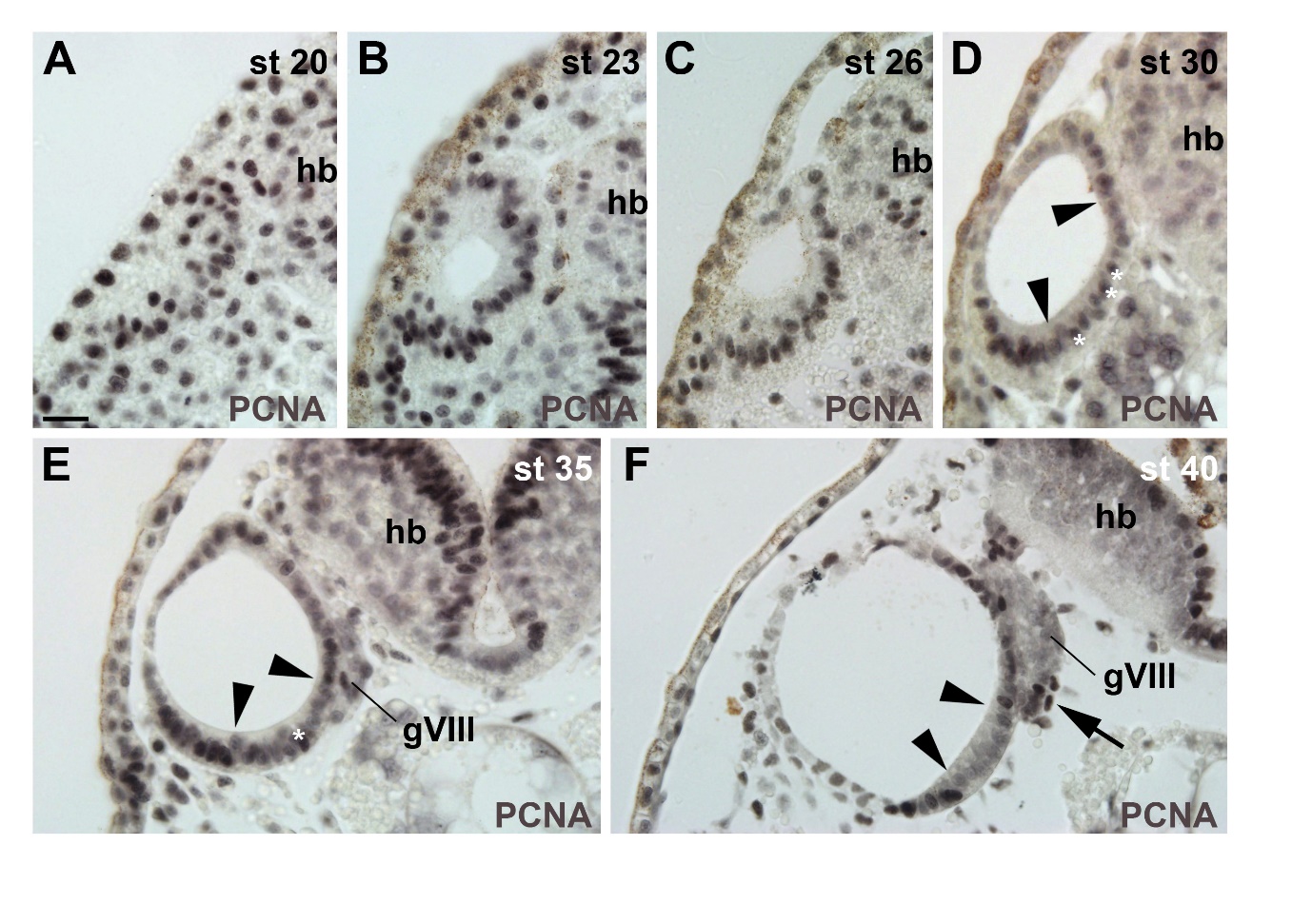
**

**Suppl. Fig. 1**

**Time course of proliferation in the otic vesicle.**

Distribution of PCNA-immunopositive, proliferative cells in transverse sections through the center of the left otic vesicle of *Xenopus* embryos from stage 20 to 40 (dorsal to the top, medial to the right). gVIII: vestibulocochlear ganglion; hb: hindbrain. Invagination of the otic vesicle is completed between stage 26 and 30. From stage 30 on, PCNA staining becomes reduced on the ventromedial side of the otic vesicle (between arrowheads) with only a few cells in this domain retaining high PCNA-levels (asterisks). PCNA-positive cells in the vestibulocochlear ganglion (shown here at stages 35 and 40) are confined to its periphery, in particular on its ventral side (arrow). ­Scale bar in **A**: 25 μm (for all panels).


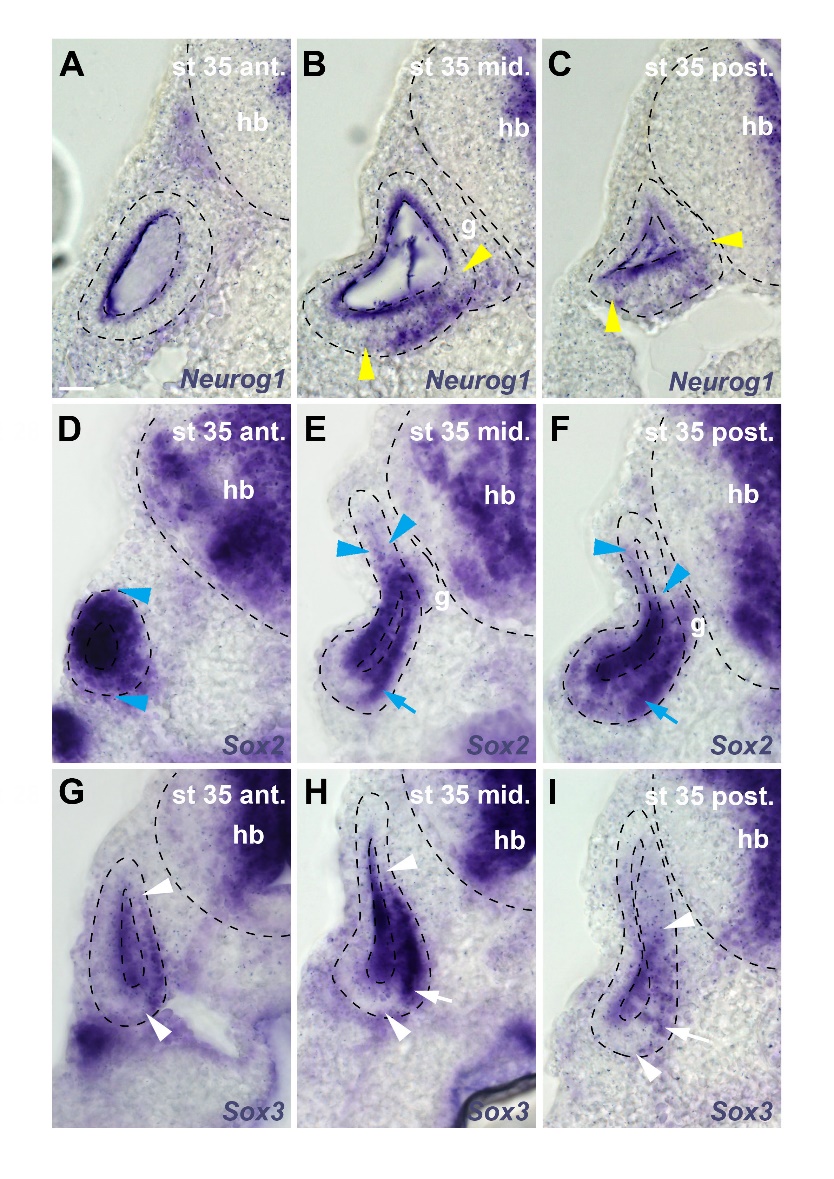


**Suppl. Fig. 2**

**Distribution of neurogenic markers at different levels of the otic vesicle at stage 35**

Distribution of *Neurog1, Sox2* and *Sox3* mRNAs in the left otic vesicle of stage 35 *Xenopus* embryos showing three approximately equidistant transverse vibratome sections (**A-I**). Arrowheads indicate extent of region containing cells expressing *Neurog1* (yellow), *Sox2* (blue) and Sox3 (white). g: vestibulocochlear ganglion (outlined with hatched lines); hb: hindbrain. Levels: ant.: anterior; mid.: midline; post.: posterior. Staining along the luminal surface and in the lumen of the otic vesicle is an artefact (trapping during in situ hybridization). **A-C**: In the anterior otic vesicle, *Neurog1* expression has declined, while in the posterior otic vesicle it has shifted to a slightly more ventral position. *Neurog1* continues to be expressed in the vestibulocochlear ganglion. **G-H**: *Sox2* continues to be expressed very broadly in the otic vesicle, being absent only from its dorsal part. It is also weakly expressed in the distal part of the vestibulocochlear ganglion. **I-L**: *Sox3* expression (which mimics the distribution of Sox3 protein; see Suppl. Fig. 5) remains confined to the ventromedial otic epithelium. Both Sox2 (**E, F**) and Sox3 (**H, I**) show relatively stronger expression in the basal part of the ventromedial otic epithelium (blue and white arrows, respectively), probably corresponding to the layer of supporting cells in the developing sensory areas. Scale bar in **A**: 25 μm (for all panels).


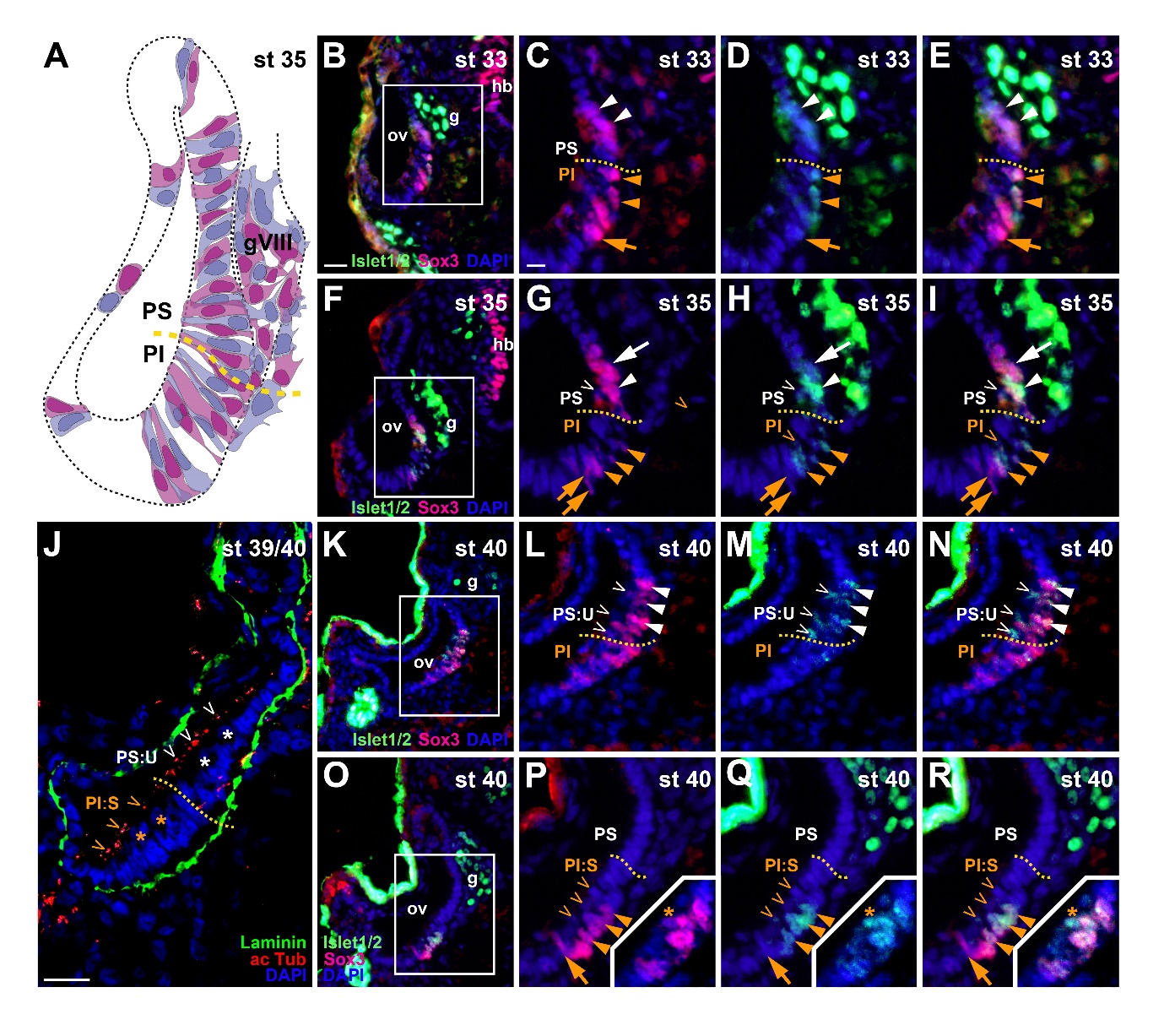


**Suppl. Fig. 3**

**Dorso-ventral separation of the sensorineural area of the otic vesicle during stages 33-40.**

**A**: Overview of cell distribution in stage 35 otic vesicle based on reconstructions from mGFP staining of a confocal z-stack (see Fig. 4). Hatched yellow line indicates approximate border between upper (PS) and lower (PI) part of the otic vesicle. **B-I, K-R**: Distribution of Sox3-immunopositive sensorineural progenitors and Islet1/2-immunopositive cells in transverse sections through the center of the left otic vesicle of *Xenopus* embryos from stage 33 to 40 (dorsal to the top, medial to the right). Overviews shown in **B** (stage 33), **F** (stage 35), **K** (stage 40, anterior of center), **O** (stage 40, posterior of center) with details of boxed areas shown in adjacent panels. DAPI was used to label nuclei. At stage 33, the domain of Sox3-immunopositive cells in the ventromedial part of the otic epithelium has separated into an upper domain (located in the pars superior of the otic vesicle: white arrows/arrowheads) and a lower domain (located in pars inferior: orange arrows/arrowheads). A subset of Sox3-positive cells in the otic epithelium shows weak Islet1/2 staining (arrowheads), whereas other Sox3-positive cells do not express Islet1/2 (arrows). At stage 40, Sox3-Islet1/2 double stained cells (arrowheads) appear to form a layer of supporting cells located basal to a layer of putative hair cells (open arrowheads), which are not immunopositive for Sox3 and mostly lack Islet1/2 as well. Inserts in **P-R** show adjacent section with one putative hair cell (asterisk) expressing Islet1/2 but not Sox3, suggesting that Sox3 is downregulated before Islet1/2 in these cells. **J**: Distribution of hair cells as revealed by immunostaining of kinocilia with acetylated tubulin at stage 39/40 (open arrowheads; asterisks indicate nuclei of hair cells). Kinocilia identified by large red dots; small red dots probably represent primary cilia. The utricular macula (U) in the pars superior (PS) can be distinguished from the saccular macula (S) in the pars inferior (PI). g: vestibulocochlear ganglion; hb: hindbrain, PS: pars superior; PI: pars inferior; S: saccular macula; U: utricular macula). **F** shows same section as Fig. 7 G-I.­ **O** shows same section as Fig. 7 J-L.­ Scale bars: **B**: 25 μm (for **B, F, K, O**). **C**: 10 μm (for **C-E**, **G-I**, **L-N**, **P-R**). **J**: 25 μm.


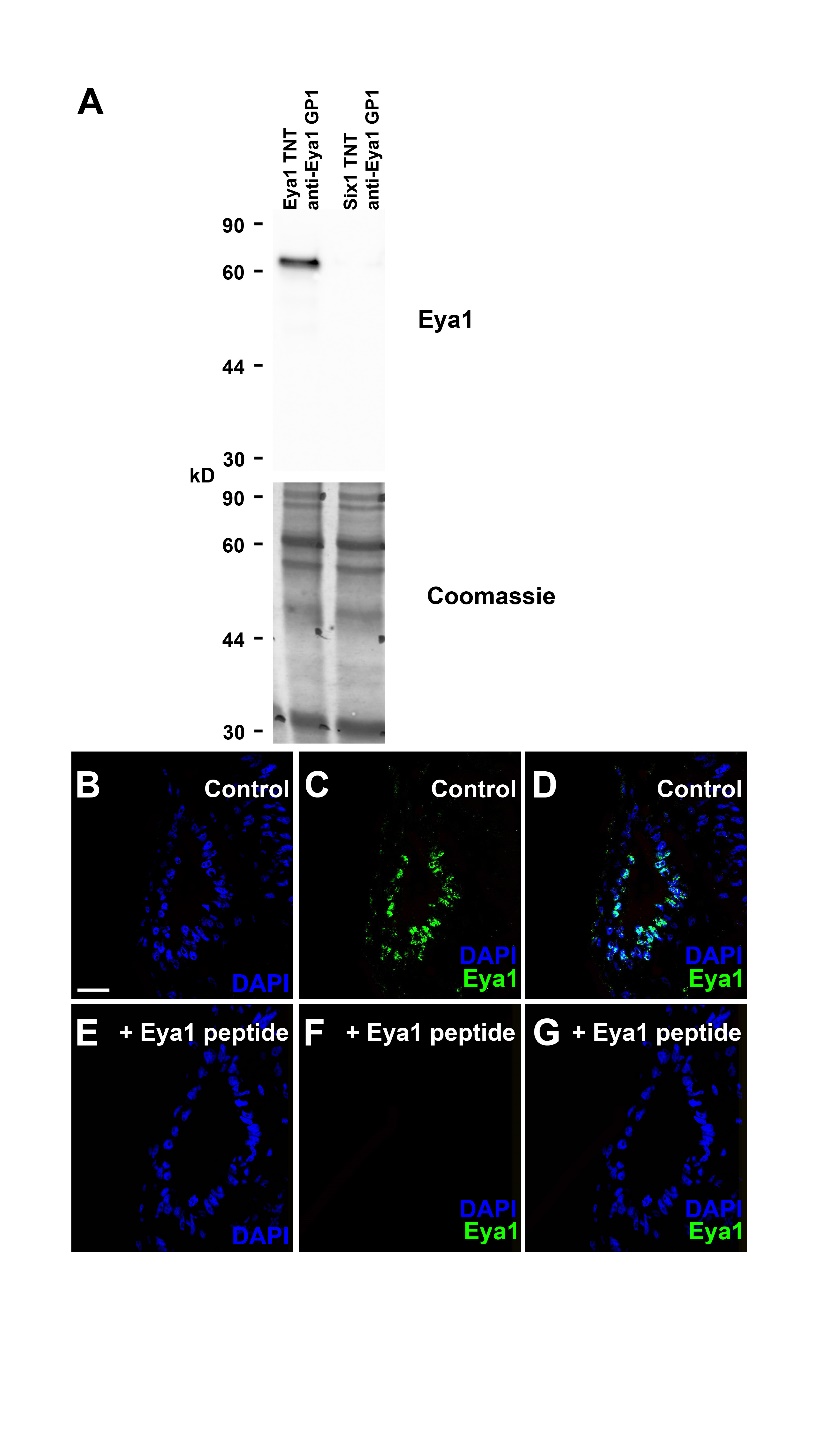


**Suppl. Fig. 4**

**Specificity of Eya1 antibodies.**

**A**: Western blot to evaluate specificity of antibody anti-Eya1 GP1 raised in guinea pigs (GP). The antibody recognizes the Eya1 protein produced by in vitro transcription and translation (TNT) of *Eya1* plasmids (left lane) but does not cross react with proteins in TNT reactions that do not contain *Eya1* (right lane: Six1 TNT). Coomassie staining demonstrates equal loading of both lanes. **B-G**: Peptide competition assay for anti-Eya1 GP1 antibody. Transverse sections through the left otic vesicle of a *Xenopus* embryos at stage 26 analyzed in single confocal planes (dorsal to the top, medial to the right). DAPI was used to label nuclei. Different channels of same section shown in **B-D** and **E-G**. Eya1 immunostaining as evident in control embryos (**B-D**) is blocked after addition of Eya1 peptide (5 μg peptide/1 μg Eya1 antibody; **E-G**). ­Scale bar in **B**: 25 μm (for **B-G**).

**
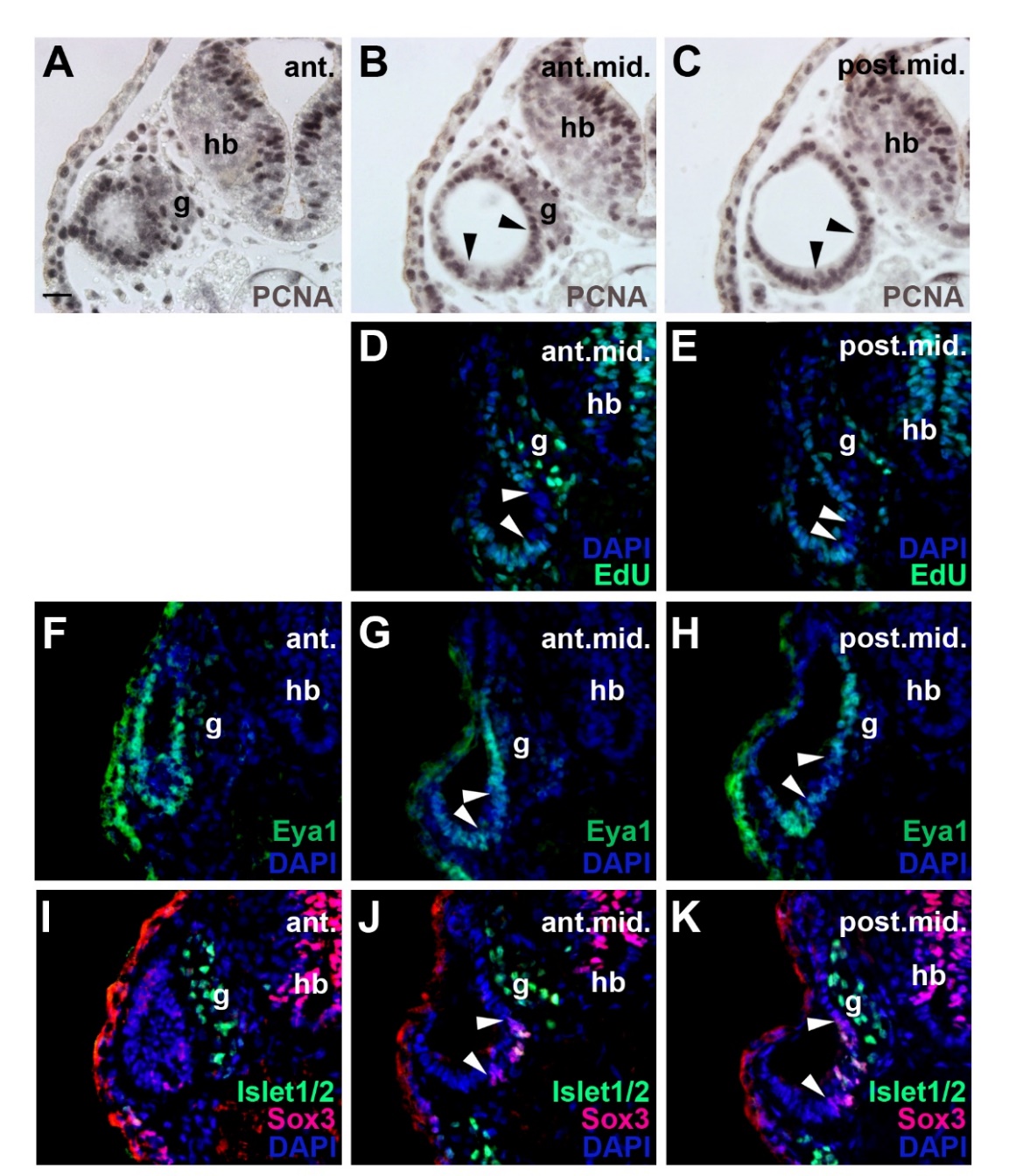
**

**Suppl. Fig. 5**

**Distribution of Eya1 in relation to progenitor and differentiation markers at different levels of the otic vesicle at stage 35.**

Distribution of proliferation markers (**A-C**: PCNA; **D, E**; EdU), Eya1-immunopositive cells (**F-H**), as well as Sox3-immunopositive sensorineural progenitors and Islet1/2-immunopositive differentiating neurons (**I-K**) in transverse sections in three approximately equidistant transverse sections of the left otic vesicle of a stage 35 *Xenopus* embryo (dorsal to the top, medial to the right). DAPI was used to label nuclei. Note the decline of proliferation and Eya1 immunostaining in the ventromedial region (between arrowheads), where Sox3-immunopositive cells are located. g: vestibulocochlear ganglion; hb: hindbrain. Levels: ant.: anterior; ant.mid.: anterior of midline; post.mid.: posterior of midline. Note that shape of the otic vesicle is better preserved in PCNA stained sections (**A-C**) due to Bouin fixation than in sections stained for Eya1, Sox3, or Islet1/2, which were fixed with PFA (**D-I**). **B** shows same section as Suppl. Fig. 1 E.­ ­**G** shows same section as Fig. 8 I, J.­ Scale bar in **A**: 25 μm (for all panels).

**
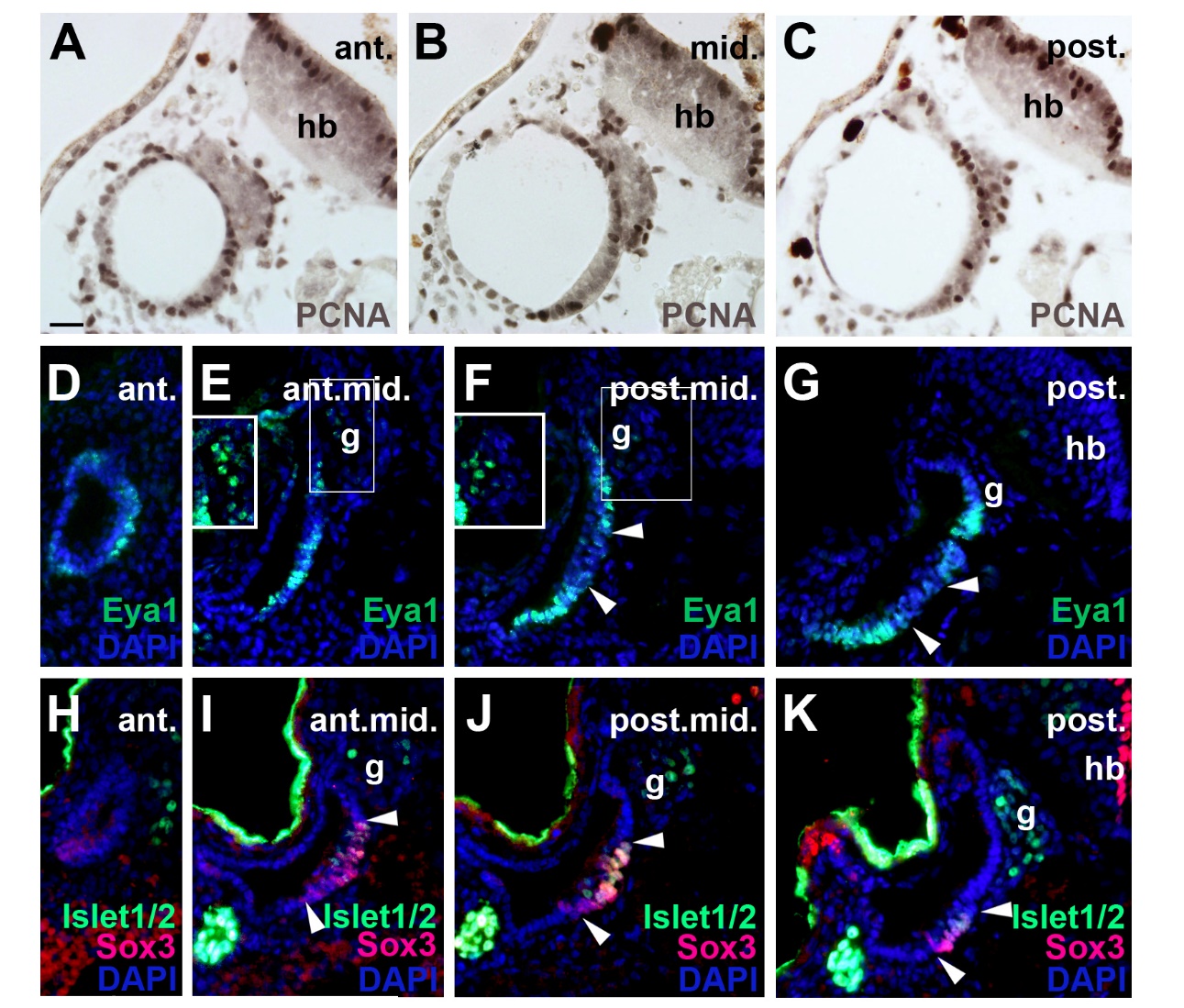
**

**Suppl. Fig. 6**

**Distribution of Eya1 protein at different levels of the otic vesicle at stage 40.**

Distribution of PCNA-immunopositive proliferation markers (**A-C**), Eya1-immunopositive cells (**D-G**), as well as Sox3-immunopositive sensorineural progenitors and Islet1/2-immunopositive differentiating neurons (**H-K**) in transverse sections in three (**A-C**) or four (**D-K**) approximately equidistant transverse sections of the left otic vesicle of a stage 40 *Xenopus* embryo (dorsal to the top, medial to the right). For PCNA, one section is shown through the midline of the otic vesicle (**B**), whereas for the other markers two sections – one slightly anterior (**E, I**) and one slightly posterior of the midline (**F, J**) – are shown. DAPI was used to label nuclei. Note the decrease of proliferation and Eya1 immunostaining in the ventromedial region (between arrowheads), where Sox3-immunopositive cells are located. G: vestibulocochlear ganglion; hb: hindbrain. Levels: ant.: anterior; ant.mid.: anterior of midline; mid.: midline; post.mid.: posterior of midline; post.: posterior. Note that shape of the otic vesicle is better preserved in PCNA stained sections (**A-C**) due to Bouin fixation than in sections stained for Eya1, Sox3, or Islet1/2, which were fixed with PFA (**D-I**). ­**B** shows same section as Suppl. Fig. 1 F.­ **F** shows same section as Fig. 8 K, L.­ **K** shows same section as Fig. 7 J-L.­ Scale bar in **A**: 25 μm (for all panels).

**
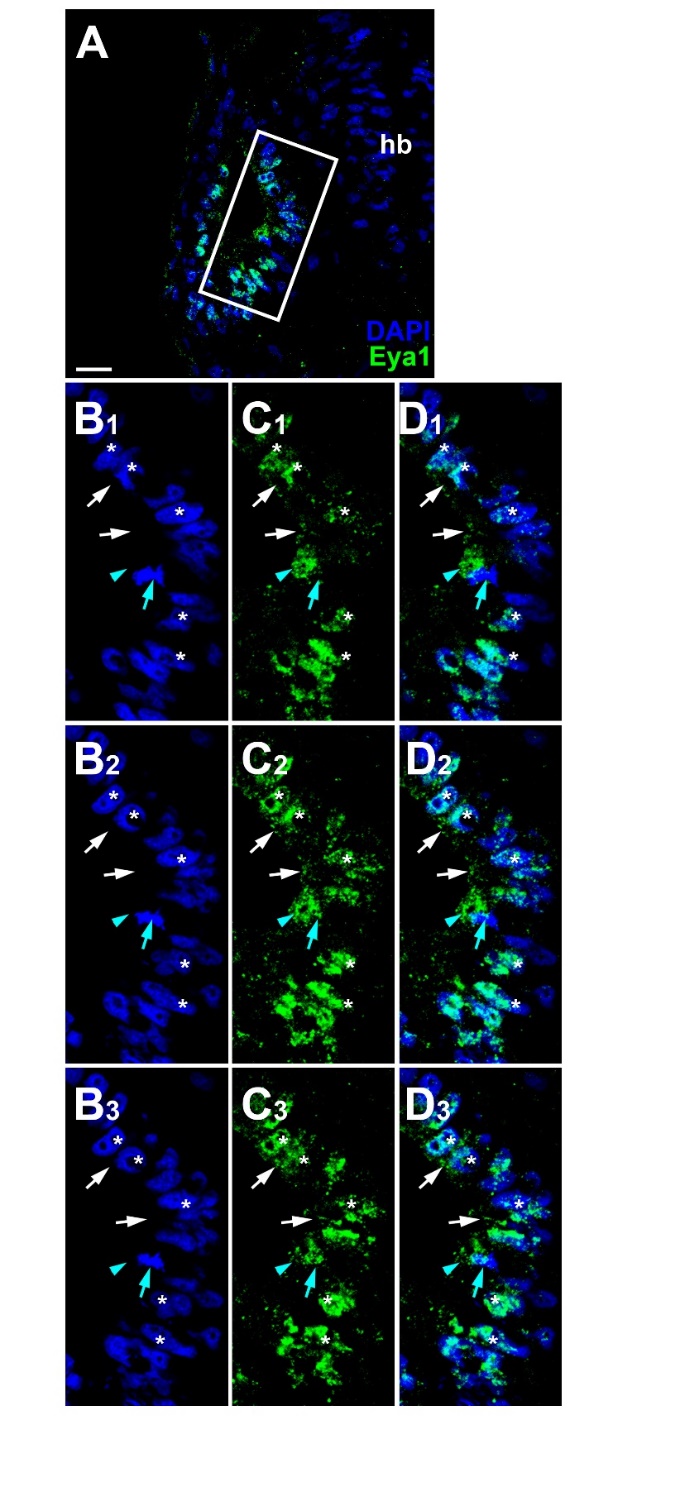
**

**Suppl. Fig. 7**

**Subcellular localization of Eya1 protein in otic vesicle and vestibulocochlear ganglion at stage 26.**

Immunostaining for Eya1 in a transverse section through the center of the left otic vesicle of *Xenopus* embryos at stage 26 analyzed by confocal microscopy (dorsal to the top, medial to the right). DAPI was used to label nuclei. **A**: Overview showing the same confocal plane as **B_2_-C_2_**. **B-D**: Magnified views of the boxed area shown in different channels (columns B-D) and in three different confocal planes (rows 1-3; 0.2 μm between adjacent planes). White asterisks show nuclear Eya1 staining, while white arrows show cytoplasmic staining in the otic epithelium. Mint arrowheads indicate cytoplasmic Eya1 staining in a dividing cell of the otic epithelium with the mint arrow indicating the division plane. Note that Eya1 shows mostly nuclear but also some cytoplasmic localization in the otic epithelium. Hb: hindbrain. ­Scale bar in **A**: 25 μm.


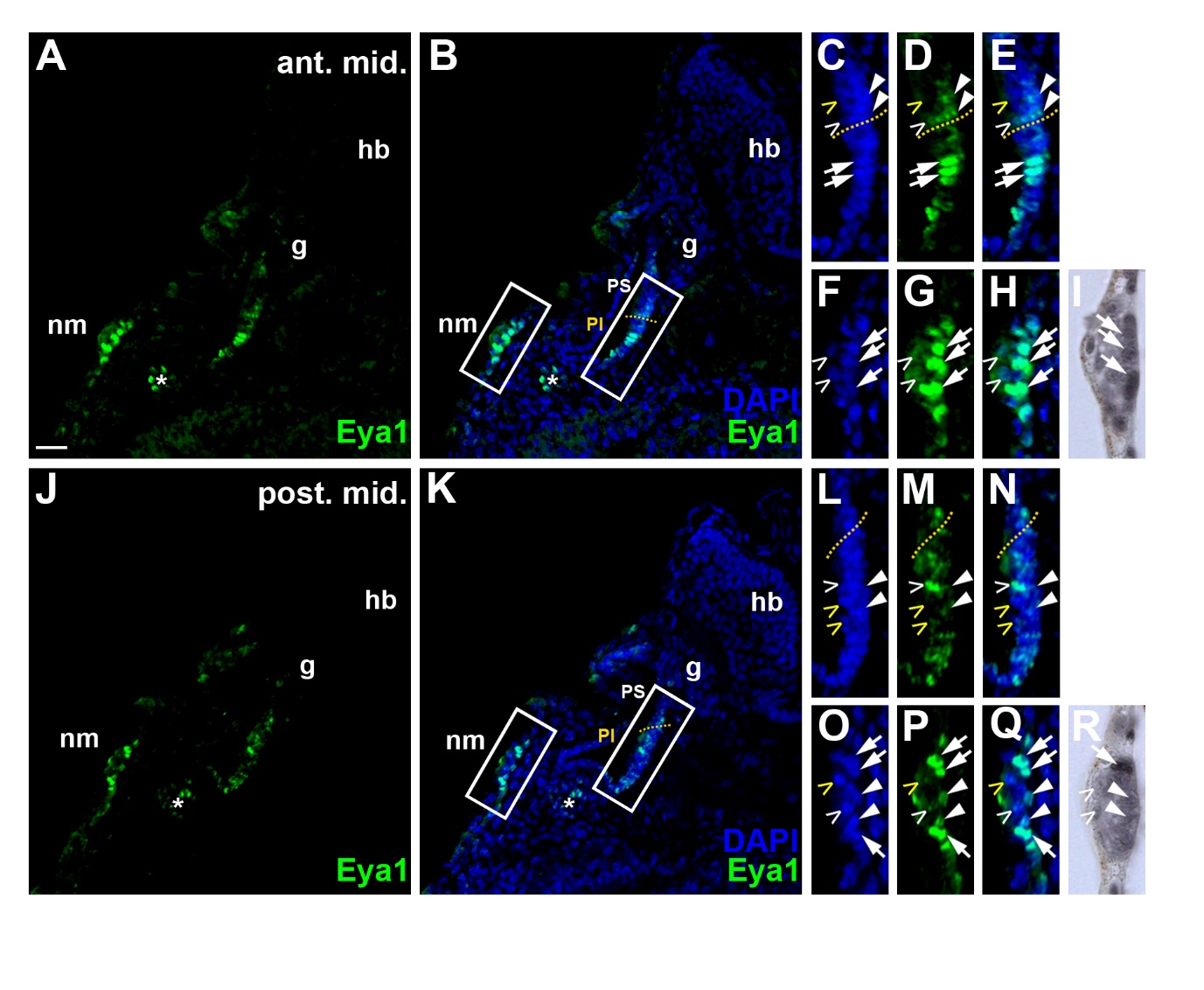


**Suppl. Fig. 8**

**Subcellular localization of Eya1 protein in otic vesicle and vestibulocochlear ganglion at stage 40.**

Immunostaining for Eya1 in transverse sections anterior (ant.mid., **A-H**) and posterior of the midline (post. mid., **J-Q**) of the left otic vesicle of *Xenopus* embryos at stage 40 analyzed by confocal microscopy (dorsal to the top, medial to the right). DAPI was used to label nuclei. Overviews of single confocal planes shown in different channels in **A, B** and **J, K**. The approximate dividing line between pars superior (PS) and pars inferior (PI) is shown by a hatched orange line. Otic epithelium and a lateral line neuromast are boxed in **B** and **K** and shown in magnified views in different channels to the right (upper panels: otic epithelium; lower panels: neuromast). Section is through the periphery of the neuromast in **F-H** and through its center in **O**-**Q**. **I** and **R** show PCNA staining through the periphery and center of a neuromast, respectively. Open arrowheads indicate hair cells, filled arrowheads indicate supporting cells and arrows indicate other, highly proliferative progenitor cells. Eya1 immunopositive hair cells are indicated by white open arrowheads, other hair cells by yellow arrowheads. Note that Eya1 is strongly expressed in nuclei of progenitors and maintained at weak levels in supporting cells, while only a subset of hair cells express Eya1 very weakly, suggesting that it is downregulated in these cells. Asterisk indicates the ganglion of the glossopharyngeal and middle lateral line nerve. g: vestibulocochlear ganglion; hb: hindbrain; nm: neuromast. ­Scale bar in **A**: 25 μm (for **A, B, J**, **K**).

**
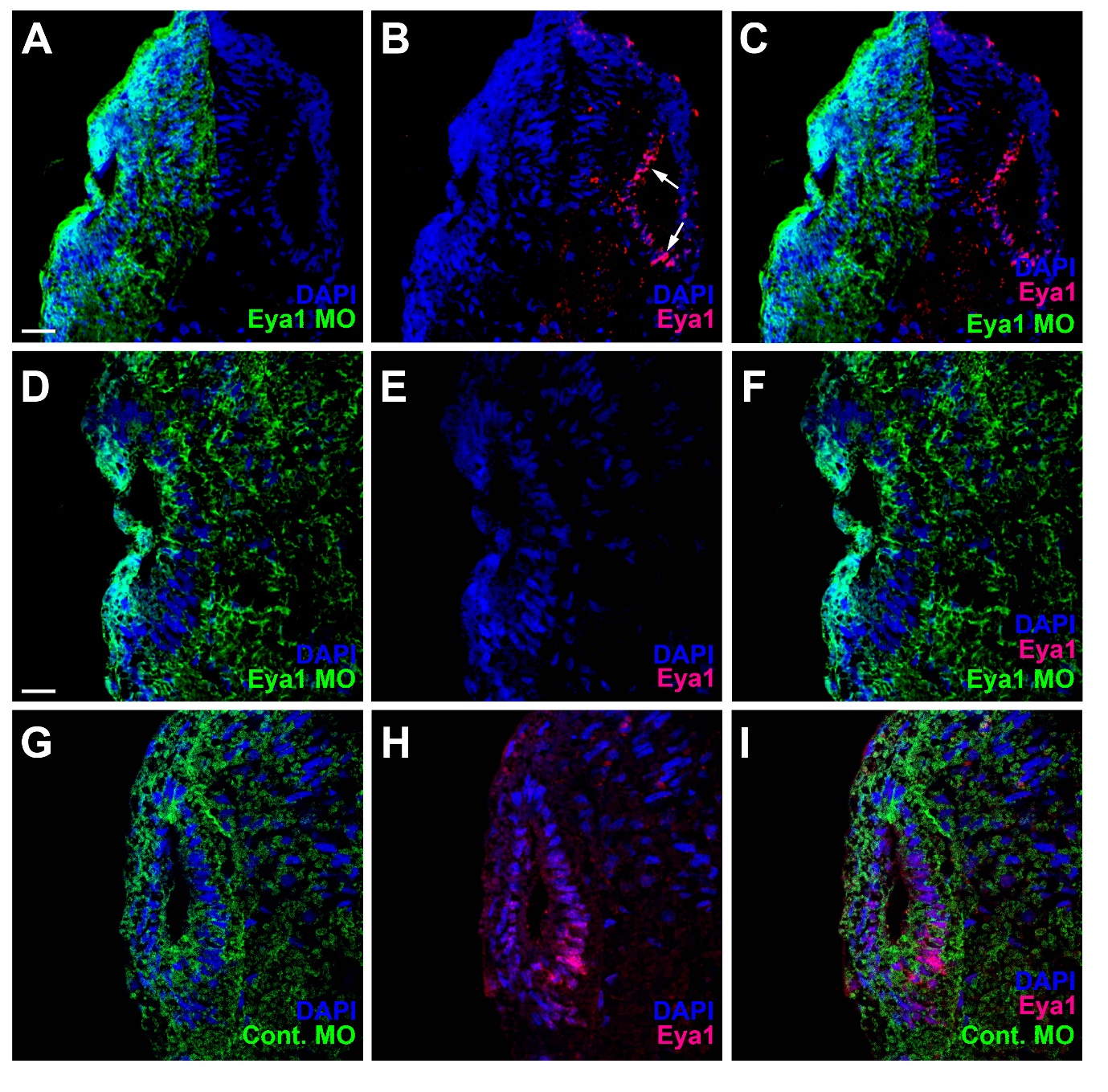
**

**Suppl. Fig. 9**

**Effective reduction of otic Eya1 immunostaining by Eya1 morpholinos**

**A-F**: Transverse section through the central otic vesicles of a stage 26 *Xenopus* embryo injected with Eya1 MO (dorsal to the top, medial to the right). DAPI was used to label nuclei. Different channels are shown in the three columns. In **A-C** the injected side is on the left, the uninjected side on the right. **D-F** show higher magnification views of the injected side. **G-I** show magnified otic vesicles in embryos injected with control MOs. Note that in Eya1 MO-injected embryos (**A-F**), Eya1-immunopositive cells, which are clearly visible in the otic vesicle on the uninjected side (arrows), are absent from the otic vesicle on the injected side suggesting that Eya1 MOs completely block Eya1 protein synthesis, while control MOs have no effect (**G-I**). ­Scale bars: **A**: 50 μm (for **A-C**); **D**: 25 μm (for **D-I**).


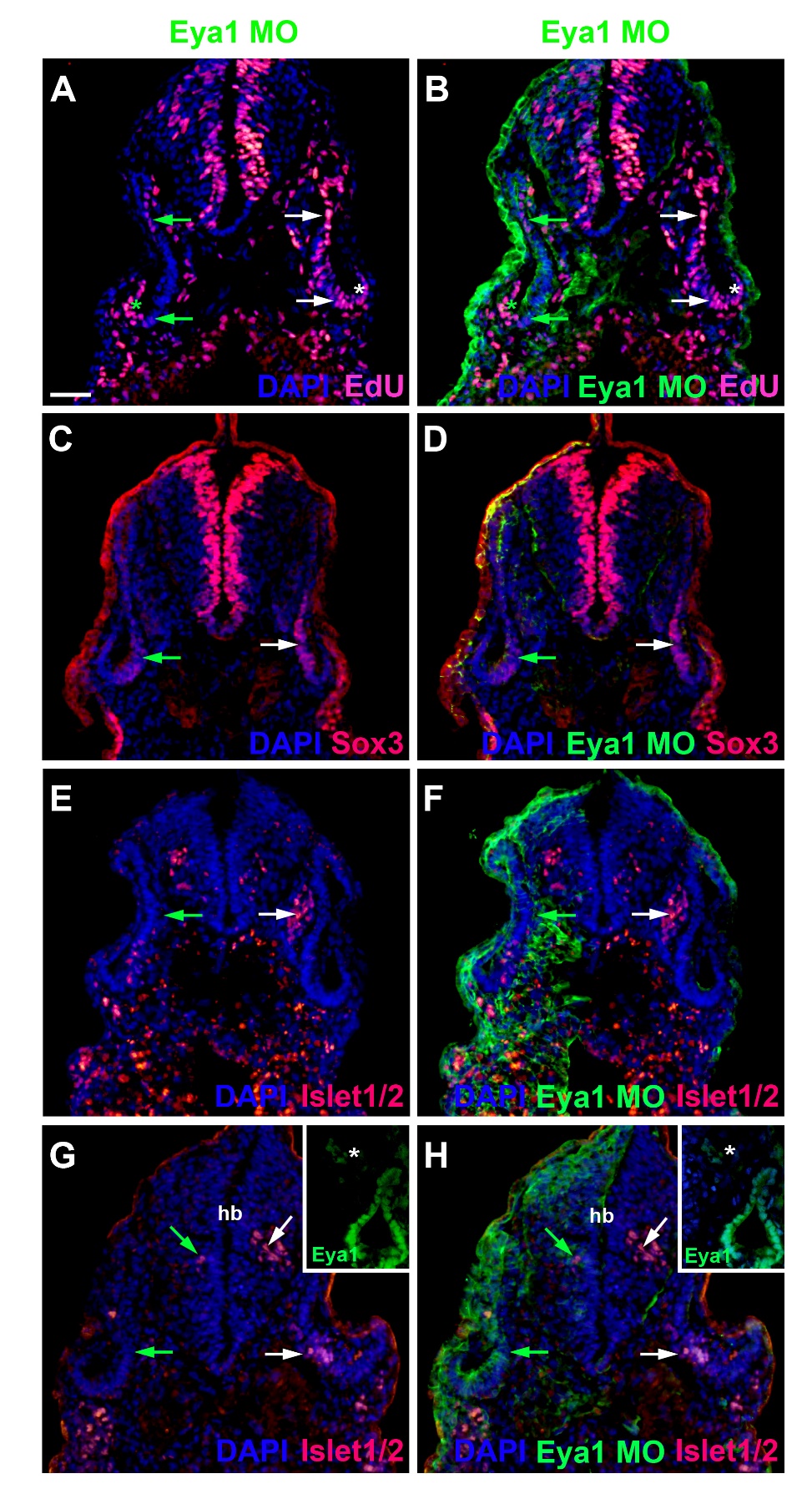


**Suppl. Fig. 10**

**Role of Eya1 for otic neurogenesis in embryos injected with Eya1 MOs: Comparison of injected and uninjected sides**

Changes of EdU-positive proliferative progenitors (**A, B**), and Sox3- (**C, D**) and Islet1/2-immunopositive cells (**E-H**) in transverse sections through the central otic vesicles of stage 35 *Xenopus* embryos injected with Eya1 MO (dorsal to the top, medial to the right). DAPI was used to label nuclei. Different channels of the same section are shown in the first and second column. In each panel, the injected side is on the left and the uninjected side is on the right. Reductions of EdU labelling and Sox3- or Islet1/2- immunoreactive cells in otic vesicle of Eya1 MO injected embryos indicated by green arrows (compare to white arrows for otic vesicle on uninjected side of same embryos). Residual EdU labelling in otic vesicle of Eya1 MO injected embryo indicated by green asterisk (compare to white asterisk for otic vesicle on uninjected side of same embryos). **E, F** and **G, H** ­show sections through two different embryos, one showing reduction of Islet1/2 in the vestibulocochlear ganglion (**E, F**), the other showing Islet1/2 reduction in the otic epithelium and the motorneurons of the hindbrain (**G, H**). The latter also are Eya1-immunopositive as shown in the insets (marked by asterisks). Scale bar in **A**: 50 μm (for all panels).


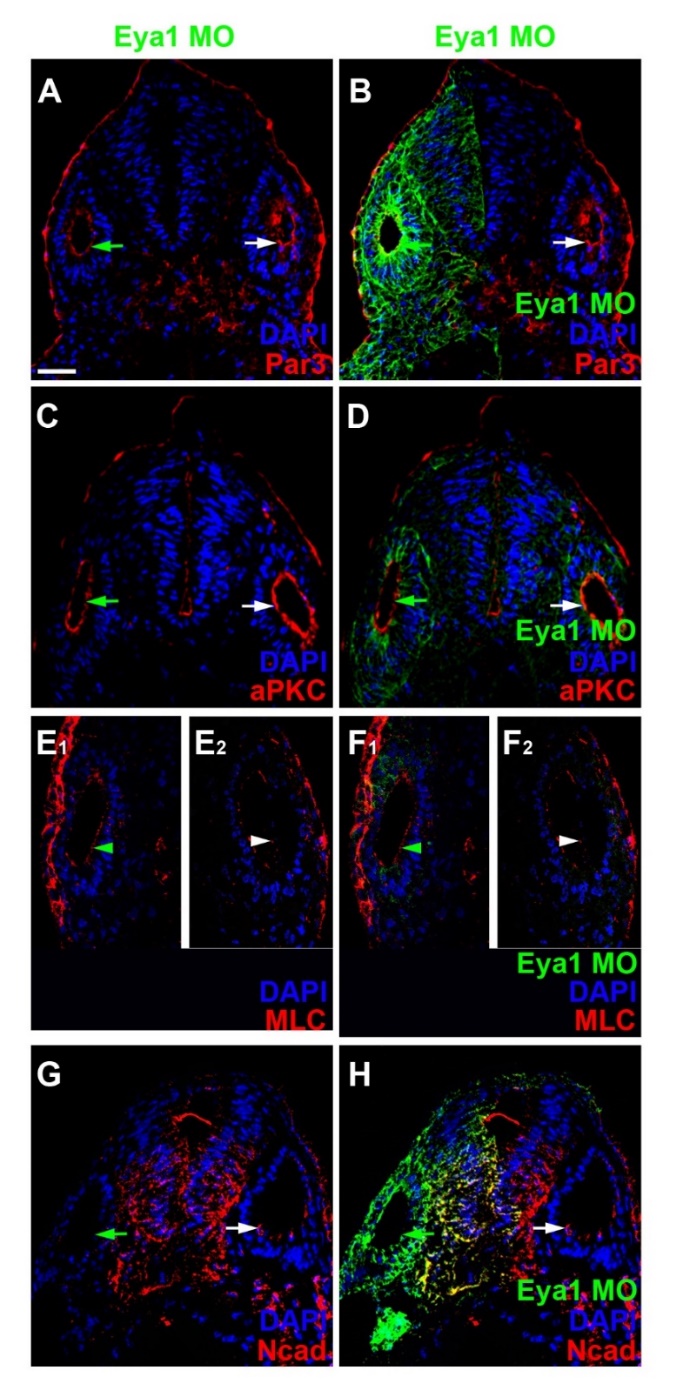


**Suppl. Fig. 11**

**Role of Eya1/Six1 for otic cell polarity in embryos injected with Eya1 MOs: Comparison of injected and uninjected sides**

Changes of Par3- (**A, B**) aPKC- (**C, D**) MLC- (**E, F**) and N-Cadherin- (**G, H**) immunostaining in transverse sections through the central otic vesicle of stage 26 *Xenopus* embryos injected with Eya1 (dorsal to the top, medial to the right). DAPI was used to label nuclei. Different channels of the same section are shown in the first and second column. In each panel, the injected side is on the left and the uninjected side is on the right. Note that Par3, aPKC and MLC remain apically localized after Eya1 MO injection, although apical protein levels of Par3 and aPKC (green arrows) but not MLC (green arrowhead) are often reduced compared on the injected side compared to the uninjected side of the same embryo (white arrows and arrowhead). However, apicolateral staining of N-cadherin is completely abolished after Eya1 MO injections (green arrow) on the injected side (compare to uninjected side, white arrow). ­Scale bar in **A**: 50 μm (for all panels).


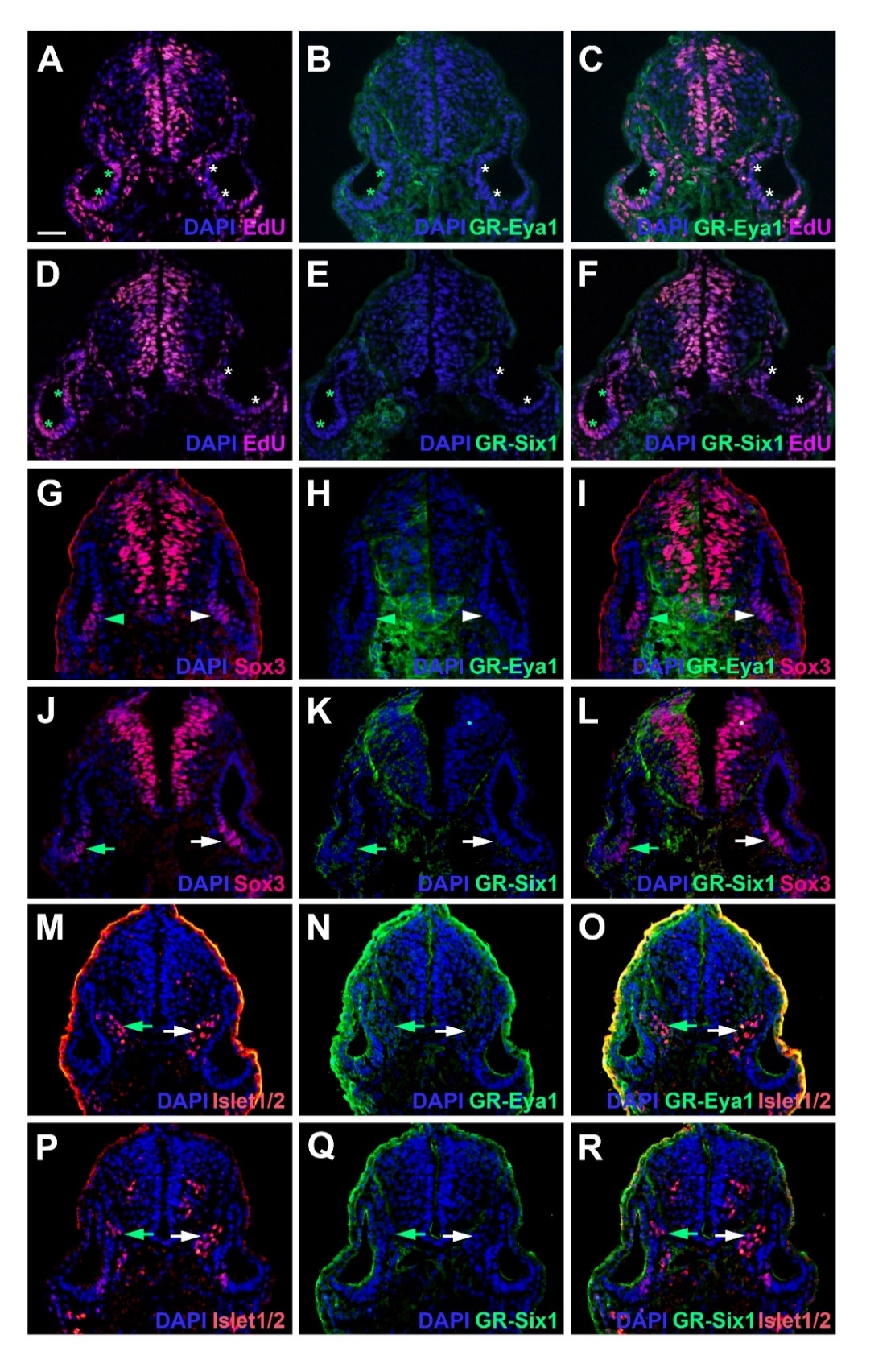


**Suppl. Fig. 12**

**Role of Eya1/Six1 for otic neurogenesis as revealed by overexpression of GR-Eya1 or GR-Six1**

Changes of EdU-positive proliferative progenitors (**A-F**), and Sox3- (**G-L**) and Islet1/2-immunopositive cells (**M-R**) in transverse sections through the central otic vesicles of stage 28 (Sox3) and 35 (EdU, Islet1/2) *Xenopus* embryos injected with GR-Eya1 (**A-C**, **G-I**, **M-O**) or GR-Six1 (**D-F**, **J-L**, **P-R**) and DEX-induced at stage 16-18 (dorsal to the top, medial to the right). DAPI was used to label nuclei. Different channels of the same section are shown in the first, second and third column. In each panel, the injected side is on the left and the uninjected side is on the right. Increased EdU labelling in the otic vesicle on the injected side (after GR-Eya1 or GR-Six1 injection) is indicated by green asterisks (compare to white asterisk for otic vesicle on uninjected side). Reductions of Sox3- (after GR-Six1 injection) and Islet1/2-immunoreactive cells (after GR-Eya1 and GR-Six1 injection) in otic vesicle on the injected side are indicated by green arrows (compare to white arrows for otic vesicle on uninjected side of same embryos). In other embryos (not shown), numbers of Islet1/2-immunopositive cells were slightly increased after GR-Six1 injection. ­Scale bar in **A**: 50 μm (for all panels).

**
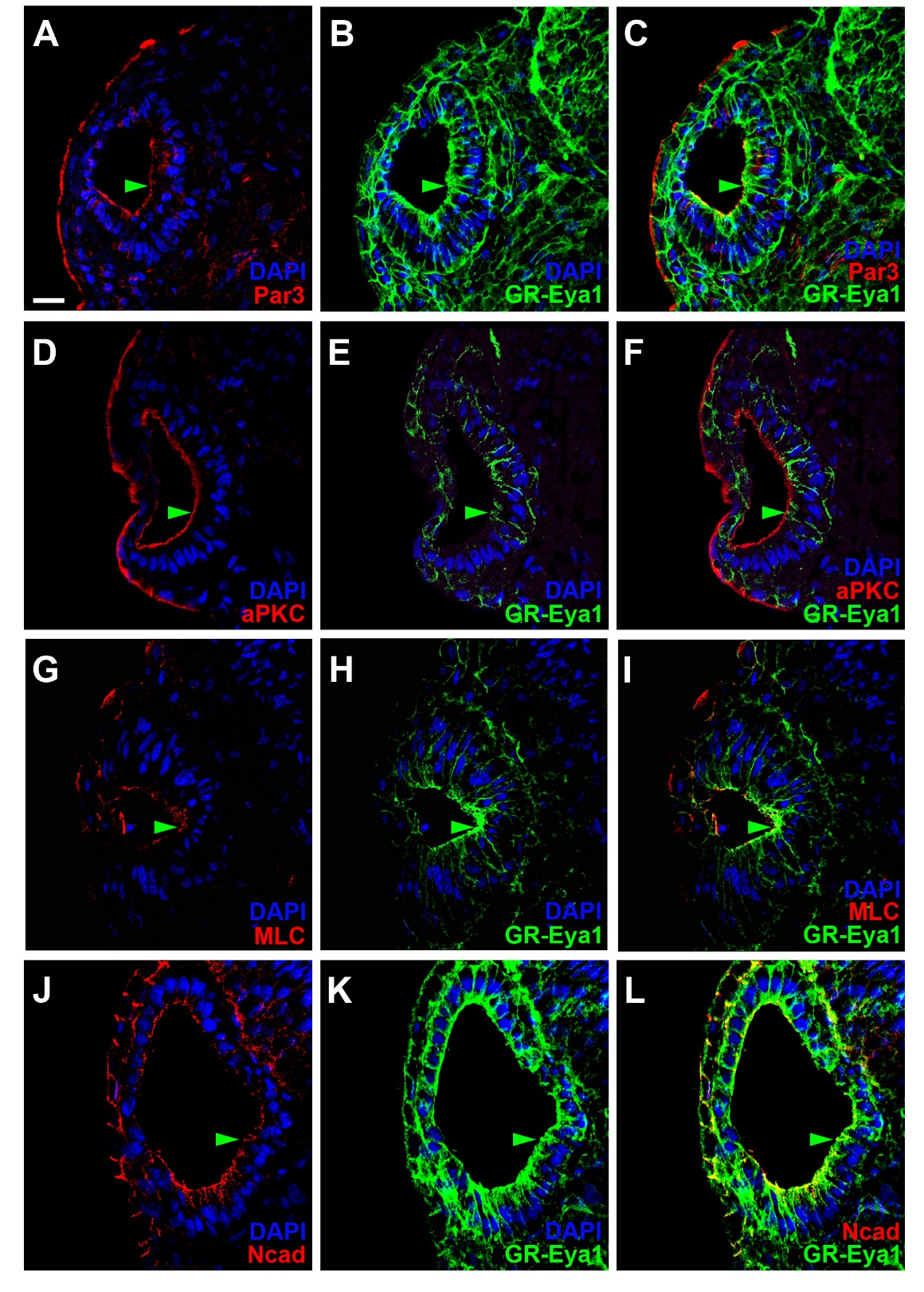
**

**Suppl. Fig. 13**

**Role of Eya1 for otic cell polarity as revealed by overexpression of GR-Eya1**

Changes of Par3- (**A-C**), aPKC- (**D-F**) MLC- (**G-I**) and N-Cadherin- (**J-L**) immunostaining in transverse sections through the central otic vesicle of stage 26 *Xenopus* embryos injected with GR-Eya1 and DEX-induced at stage 16-18 (dorsal to the top, medial to the right). DAPI was used to label nuclei. Different channels of the same section are shown in the first, second and third column. There are no major changes in protein distribution compared to uninjected embryos (see Fig. 2). Apical or apicolateral localization of all markers highlighted by green arrowheads. ­Scale bar in **A**: 25 μm (for all panels).
