# Supplementary material for: Otic Neurogenesis in Xenopus laevis: Proliferation, Differentiation, and the Role of Eya1
Source: Front Neuroanat. 2021 Sep 20;15:722374. doi: 10.3389/fnana.2021.722374 (PMC8488300; doi:10.3389/fnana.2021.722374)
Supplement: Supplementary file 2 [file Data_Sheet_2.docx]

**Otic neurogenesis in *Xenopus laevis*:**

**Proliferation, differentiation, and the role of Eya1**

**Supplemental material**

**Supplemental Tables**

| **Suppl. Table 1**  **Percentage of mitotic (pH3 positive) nuclei during otic vesicle development** | | | |
| --- | --- | --- | --- |
| **Embryos** | **DAPI nuclei** | **PH3 nuclei** | **%** |
|  |  |  |  |
| **St 26, embryo 1** | 317 | 7 | 2.208 |
| **St 26, embryo 2** | 358 | 5 | 1.396 |
| **St 26, embryo 3** | 466 | 6 | 1.28 |
| **St 35, embryo 1** | 798 | 22 | 2.756 |
| **St 35, embryo 2** | 727 | 21 | 2.888 |
| **St 35, embryo 3** | 741 | 16 | 2.159 |

| **Suppl. Table 2**  **Percentage of mitotic (pH3 positive) nuclei during otic vesicle development**  **after Eya1 gain and loss of function** | | | |
| --- | --- | --- | --- |
| **Embryos** | **DAPI nuclei** | **PH3 nuclei** | **%** |
|  |  |  |  |
| **St 26, GR-Eya1 inj., embryo 1** | 375 | 51 | 13.6 |
| **St 26, GR-Eya1 inj., embryo 2** | 261 | 30 | 11.49 |
| **St 26, GR-Eya1 inj., embryo 3** | 294 | 37 | 12.58 |
| **St 26, Cont. MO inj., embryo 1** | 393 | 5 | 1.2 |
| **St 26, Cont. MO inj., embryo 2** | 376 | 6 | 1.5 |
| **St 26, Cont. MO inj., embryo 3** | 268 | 8 | 2.0 |
| **St 26, Eya1 MO1+2 inj., embryo 1** | 389 | 1 | 0.25 |
| **St 26, Eya1 MO1+2 inj., embryo 2** | 371 | 1 | 0.2 |
| **St 26, Eya1 MO1+2 inj., embryo 3** | 422 | 4 | 0.9 |
